# Branched‐Chain Amino Acid Accumulation Fuels the Senescence‐Associated Secretory Phenotype

**DOI:** 10.1002/advs.202303489

**Published:** 2023-11-15

**Authors:** Yaosi Liang, Christopher Pan, Tao Yin, Lu Wang, Xia Gao, Ergang Wang, Holly Quang, De Huang, Lianmei Tan, Kun Xiang, Yu Wang, Peter B. Alexander, Qi‐Jing Li, Tso‐Pang Yao, Zhao Zhang, Xiao‐Fan Wang

**Affiliations:** ^1^ Department of Pharmacology and Cancer Biology Duke University Medical Center Durham NC 27710 USA; ^2^ State Key Laboratory of Molecular Biology Shanghai Institute of Biochemistry and Cell Biology Center for Excellence in Molecular Cell Science Chinese Academy of Sciences Shanghai 200031 China; ^3^ Children's Nutrition Research Center Department of Pediatrics Baylor College of Medicine Houston TX 77030 USA; ^4^ School of Basic Medical Sciences Division of Life Sciences and Medicine University of Science and Technology of China Hefei 230026 China; ^5^ Center for Regenerative Medicine Massachusetts General Hospital Harvard Medical School Boston MA 02114 USA; ^6^ Department of Immunology Duke University Medical Center Durham NC 27710 USA; ^7^ Institute of Molecular and Cell Biology Agency for Science Technology and Research (A*STAR) Singapore 138673 Singapore; ^8^ Singapore Immunology Network Agency for Science Technology and Research (A*STAR) Singapore 138673 Singapore

**Keywords:** age‐related inflammation, BCAA, mTORC1, SASP, senescence

## Abstract

The essential branched‐chain amino acids (BCAAs) leucine, isoleucine, and valine play critical roles in protein synthesis and energy metabolism. Despite their widespread use as nutritional supplements, BCAAs’ full effects on mammalian physiology remain uncertain due to the complexities of BCAA metabolic regulation. Here a novel mechanism linking intrinsic alterations in BCAA metabolism is identified to cellular senescence and the senescence‐associated secretory phenotype (SASP), both of which contribute to organismal aging and inflammation‐related diseases. Altered BCAA metabolism driving the SASP is mediated by robust activation of the BCAA transporters Solute Carrier Family 6 Members 14 and 15 as well as downregulation of the catabolic enzyme BCAA transaminase 1 during onset of cellular senescence, leading to highly elevated intracellular BCAA levels in senescent cells. This, in turn, activates the mammalian target of rapamycin complex 1 (mTORC1) to establish the full SASP program. Transgenic *Drosophila* models further indicate that orthologous BCAA regulators are involved in the induction of cellular senescence and age‐related phenotypes in flies, suggesting evolutionary conservation of this metabolic pathway during aging. Finally, experimentally blocking BCAA accumulation attenuates the inflammatory response in a mouse senescence model, highlighting the therapeutic potential of modulating BCAA metabolism for the treatment of age‐related and inflammatory diseases.

## Graphical abstract

Upregulation of the branched‐chain amino acid (BCAA) transporters, solute carrier Family 6 member 14/15 (SLC6A14/15), coupled with the downregulation of the catabolic enzyme BCAA transaminase 1 (BCAT1), drives the establishment of senescence‐associated secreted phenotypes (SASP) through BCAA–mammalian target of rapamycin complex 1 (mTORC1) signaling pathway in cellular senescence and promotes senescence‐related inflammatory responses in fruit flies and mice.

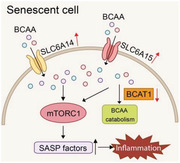

## 1 Introduction

The branched‐chain amino acids (BCAAs) valine, leucine, and isoleucine are essential amino acids containing non‐linear aliphatic side chains. Apart from their roles in protein synthesis and as metabolic precursors for the tricarboxylic acid (TCA) cycle and other pathways,^[^ ^1^ ^]^ BCAAs also function as molecular signals to activate mammalian target of rapamycin complex 1 (mTORC1),^[^ ^2^, ^3^ ^]^ a crucial regulator of cell growth and metabolism. Despite their widespread use as dietary supplements to potentially improve exercise performance and muscle growth,^[^ ^4^ ^]^ their roles in aging and inflammatory diseases are complex and remain controversial.^[^ ^5^ ^]^ While some studies suggest that BCAA supplementation may improve skeletal muscle function in middle‐aged mice,^[^ ^6^ ^]^ others suggest that high BCAA intake can impair health and lifespan.^[^ ^7^ ^]^ In addition, low BCAA intake has been associated with improved metabolic health and extended longevity in mice,^[^ ^7^, ^8^ ^]^ as well as higher survival rates and better performance in Alzheimer's disease models.^[^ ^9^ ^]^ However, because most of these previous studies were based on manipulating dietary or medium BCAA availability to investigate their potential roles in these contexts, any intrinsic alterations in BCAA metabolism that might occur during the aging process remain largely unknown.

Cellular senescence is an irreversible cell cycle arrest phenotype^[^ ^10^ ^]^ that is induced by various stresses, including DNA damage, oxidative stress, and oncogene activation.^[^ ^11^ ^]^ Senescent cells accumulate in aged and inflammatory tissues where they drive age‐associated and inflammatory phenotypes primarily through the senescence‐associated secretory phenotype (SASP).^[^ ^12^, ^13^ ^]^ Known SASP factors include various cytokines, chemokines, and proteases, which together produce a proinflammatory microenvironment promoting tissue deterioration.^[^ ^12^, ^14^ ^]^ Targeting senescence has been shown to delay the aging‐related phenotypes^[^ ^15^, ^16^, ^17^, ^18^, ^19^, ^20^ ^]^ and relieve the progression of various diseases including liver fibrosis,^[^ ^21^, ^22^ ^]^ osteoarthritis,^[^ ^23^ ^]^ and COVID‐19.^[^ ^24^, ^25^ ^]^ However, the molecular pathways that distinguish senescence from alternative cell fates such as quiescence and apoptosis, as well as those molecules responsible for establishing and maintaining the SASP, remain incompletely understood. Previous studies have shown that senescent cells undergo metabolic reprogramming,^[^ ^26^, ^27^, ^28^ ^]^ which involves broad changes in glucose,^[^ ^29^, ^30^, ^31^, ^32^, ^33^ ^]^ nucleotide,^[^ ^34^, ^35^ ^]^ and lipid metabolism.^[^ ^36^, ^37^, ^38^, ^39^ ^]^ Although some studies have revealed an altered abundance and possible functions of certain amino acids in senescent cells,^[^ ^40^, ^41^, ^42^, ^43^, ^44^, ^45^, ^46^, ^47^ ^]^ the intrinsic changes in amino acid metabolism that underlie these alterations have remained obscure, such that it remains unclear whether and how senescent cells alter amino acid metabolism and how these changes might impact senescence.

In this study, we discovered consistent upregulation of BCAA transporters and reduced BCAA catabolism in senescent cells. These intrinsic alterations lead to intracellular BCAA accumulation and mTORC1 activation in senescent cells to promote SASP establishment. Importantly, manipulating these BCAA regulators is sufficient to block SASP factor expression in vitro and in vivo. Together, these findings uncover BCAA buildup as upstream of mTORC1 in senescent cells and provide new therapeutic targets for senescence‐ and age‐related inflammatory disorders.

## 2 Results

### 2.1 Alterations in BCAA Metabolism during Cellular Senescence

**Figure 1:**
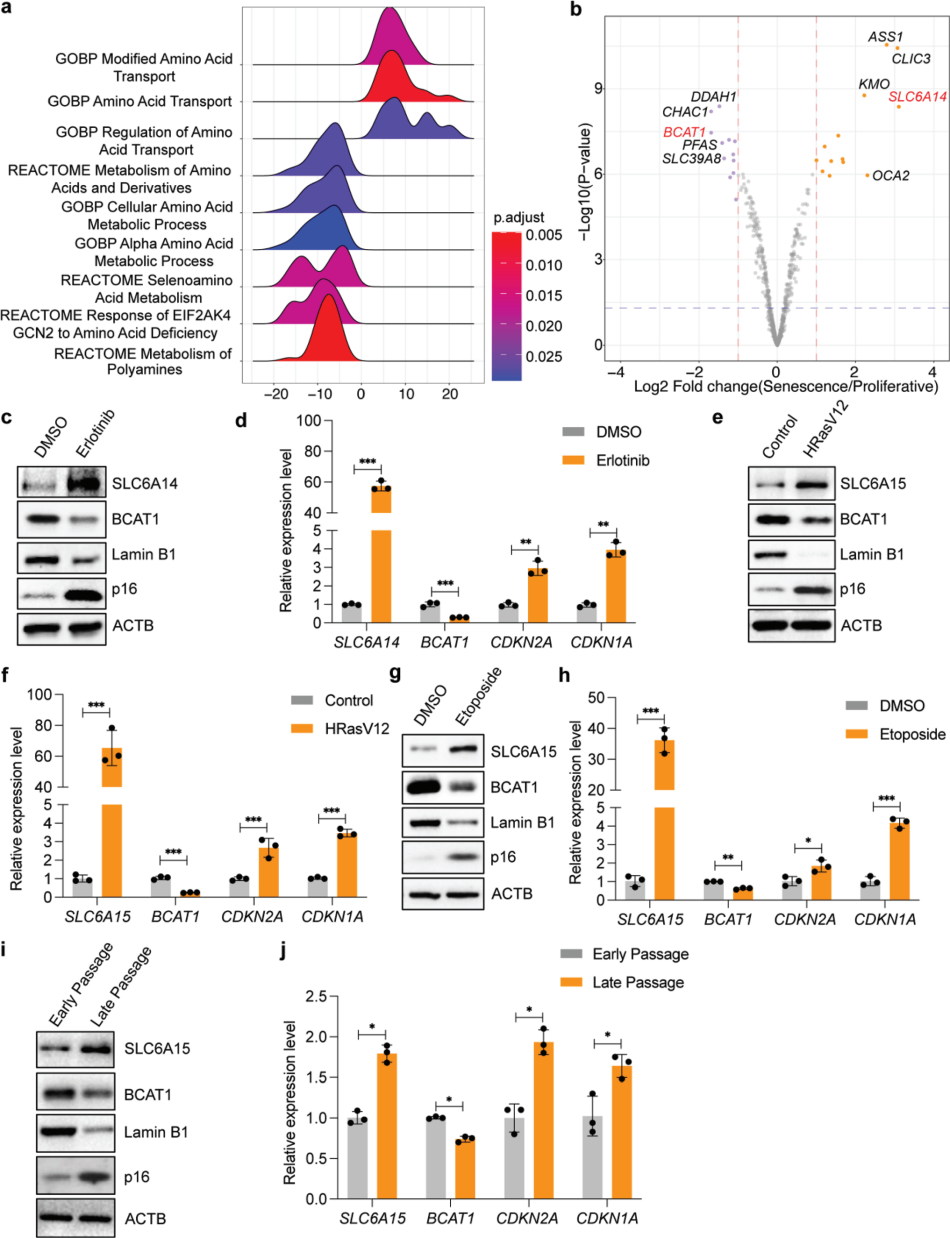
Altered BCAA metabolism in senescent cells. a) Altered amino acid metabolic pathways in senescent NHBE cells. b) Top differentially expressed genes in amino acid‐related metabolic pathways between senescent NHBE cells and proliferative cells. c) Immunoblots and d) RT‐qPCR showing increased SLC6A14 and decreased BCAT1 in senescent NHBE cells. Cells were induced to senescence by treatment with the EGFR inhibitor erlotinib (1 × 10^‐6^ m). e,f) Increased SLC6A15 and reduced BCAT1 in oncogene‐induced senescence. IMR90 cells with Tet‐on HRasV12 were treated with doxycycline (1 µg mL^‐1^) for 9 d. g,h) Increased SLC6A15 and reduced BCAT1 in DNA damage‐induced senescence. IMR90 cells were treated with 100 × 10^‐6^ m etoposide for 24 h to induce DNA damage and then cultured for another 8 d. i,j) Increased SLC6A15 and reduced BCAT1 in replicative senescence. Early (passage doubling: 38) and late passage (passage doubling: 78) IMR90 cells were collected for immunoblotting and RT‐qPCR. d,f,h,j) *n* = 3, mean ± SD, two‐tailed Student's t‐test. *P < 0.05, **P < 0.05, ***P < 0.001. SLC6A14: solute carrier family 6 member 14; CLIC3: chloride intracellular channel 3; ASS1: argininosuccinate synthetase 1; OCA2: oculocutaneous albinism II; KMO: kynurenine 3‐monooxygenase; BCAT1: branched chain amino acid transaminase 1; CHAC1: ChaC glutathione specific gamma‐glutamylcyclotransferase 1; DDAH1: dimethylarginine dimethylaminohydrolase 1; PFAS: phosphoribosylformyl‐glycinamidine synthase; SLC39A8: solute carrier family 39 member 8; SLC6A15: solute carrier family 6 member 15; CDKN1A(p21): cyclin dependent kinase inhibitor 1A; CDKN2A(p16): cyclin dependent kinase inhibitor 2A; ACTB: actin beta; HRasV12: HRas proto‐oncogene with G12V mutation.

We previously found that epidermal growth factor receptor (EGFR) signaling inhibition causes senescence of normal human bronchial epithelial (NHBE) cells in vitro and premature aging in vivo.^[^ ^22^, ^48^, ^49^, ^50^, ^51^ ^]^ Using this senescence model, we used the EGFR inhibitor erlotinib to induce senescence and conducted unbiased transcriptional profiling to characterize proliferating, quiescent, and senescent populations of NHBE cells.^[^ ^49^ ^]^ Interestingly, after confirming the previously reported reprogramming of lipid and glucose metabolism in senescent NHBE cells (Figure S1a,b, Supporting Information),^[^ ^26^, ^27^, ^28^ ^]^ we noticed significant changes in pathways related to amino acid transport and metabolism (**Figure** **1**a). Specifically, we identified the amino acid transporter Solute Carrier Family 6 member 14 (SLC6A14) as one of the most highly upregulated genes; conversely, the branched chain amino acid transaminase 1 (BCAT1) was significantly decreased upon senescence induction among the amino acid‐related metabolic pathways (Figure 1b and Figure S1c,d, Supporting Information).

SLC6A14 is a Na^+^ and Cl^−^‐dependent broad spectrum amino acids transporter that mediates the uptake of neutral and cationic amino acids, with high affinities for BCAAs.^[^ ^52^ ^]^ It is primarily expressed in epithelial cells of the digestive tract and lung to mediate nutrient uptake and fluid homeostasis.^[^ ^53^, ^54^ ^]^ BCAT1 mediates the reversible deamination of BCAA to produce branched‐chain α‐keto acids via the conversion of α‐ketoglutarate into glutamate.^[^ ^1^, ^55^ ^]^ BCAT1‐produced branched‐chain α‐keto acids can be further processed by a series of enzymes and shuttled into different metabolic pathways, including TCA cycle, lipid synthesis, and gluconeogenesis.^[^ ^1^, ^55^ ^]^ Although BCAT catalysis is theoretically reversible, animals cannot *de novo* synthesize branched‐chain α‐keto acids; thus, BCAT1 primarily catalyzes BCAA catabolism.^[^ ^1^, ^55^ ^]^ Therefore, we postulated that elevated expression of SLC6A14 and reduced BCAT1 should have interconnected impacts on senescent cell metabolism resulting in increased intracellular BCAAs.

To demonstrate this, we first validated these gene expression changes in senescent cells. Consistent with our transcriptome data, the increase of SLC6A14 and decrease of BCAT1 expression was observed in senescent epithelial cells (Figure 1c,d and Figure S2a, Supporting Information). This phenomenon is further supported by published datasets of senescent human IMR90 fibroblasts,^[^ ^56^ ^]^ one of the most commonly used cellular senescence models. In senescent IMR90 cells,^[^ ^56^ ^]^ we also observed a consistent decrease in BCAT1, although we did not detect elevated SLC6A14 expression. Instead, upon senescence induction we found an increase in SLC6A15, a family member that shares similar functions with SLC6A14. This difference is likely explained by cell type heterogeneity, since the functions of these transporters are redundant and they exhibit distinct tissue distributions.^[^ ^52^, ^53^, ^57^, ^58^, ^59^ ^]^ In support of this, the single cell type Atlas indicates that SLC6A14 is mainly expressed in epithelial and endocrine cells, while SLC6A15 is expressed in neuronal cells, melanocytes, and fibroblasts.^[^ ^60^ ^]^ To confirm this data mining, we used three independent methods to induce senescence in human IMR90 fibroblasts: overexpression of the oncogene HRasV12 (HRas proto‐oncogene with G12V mutation), treatment with the DNA‐damage inducer etoposide, and replicative exhaustion. Indeed, both immunoblotting and quantitative reverse transcription PCR (RT‐qPCR) showed that SLC6A15 is significantly upregulated and BCAT1 reduced in all three senescent fibroblast models (Figure 1e–j, Figure S2b–g, Supporting Information). Together, these results demonstrate consistently increased BCAA transporter expression and reduced BCAA transamination machinery in diverse senescent cell models.

### 2.2 Suppressing BCAA Accumulation Blocks the SASP

**Figure 2:**
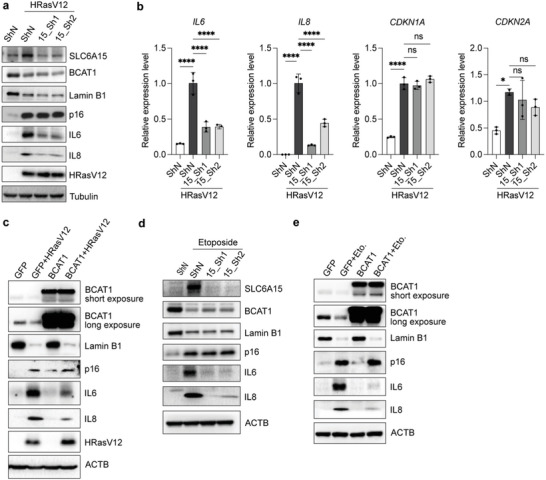
SLC6A15 and BCAT1 regulate the SASP. a,c) Immunoblots showing that a) SLC6A15 knockdown or c) BCAT1 overexpression impairs expression of IL6 and IL8 in oncogene‐induced senescence. IMR90 cells with Tet‐on HRasV12 were treated with doxycycline (1 µg mL^‐1^) for 9 d to induce senescence. Cells were infected with lentivirus to express ShN, SLC6A15 shRNA, GFP, or BCAT1, as indicated. b) RT‐qPCR analysis showing that SLC6A15 knockdown inhibits IL6 and IL8 expression in oncogene‐induced senescence. *n* = 3, mean ± SD, one‐way ANOVA test with Dunnett's multiple comparisons, *P < 0.05, ****P < 0.0001. d) SLC6A15 knockdown or e) BCAT1 overexpression impairs the expression of IL6 and IL8 in DNA damage‐induced senescence. IMR90 cells were treated with 100 × 10^‐6^ m etoposide for 24 h and cultured for 8 d before collecting samples. Cells were infected with lentivirus to express ShN, SLC6A15 shRNA, GFP, or BCAT1, as indicated. Eto.: Etoposide; IL6: interleukin 6; IL8: interleukin 8.

To assess a possible functional role for BCAA metabolism during cellular senescence, we first blocked SLC6A15 expression using short hairpin RNA and assessed senescence establishment using a variety of senescent cell models. Interestingly, SLC6A15 depletion had no effect on cell cycle arrest, as indicated by high p16 (Cyclin Dependent Kinase Inhibitor 2A) expression, reduced Lamin B1, and senescence‐associated‐beta‐galactosidase (SA‐β‐Gal) positivity in senescent IMR90 fibroblasts (**Figure** **2**a,b,d and Figure S3a,c,e, Supporting Information). However, we noticed that reduced expression of SLC6A15 significantly inhibited the production of prominent SASP factors, including interleukin 6 (IL6) and interleukin 8 (IL8) (Figure 2a,b,d). Indeed, RT‐qPCR analysis revealed that SLC6A15 depletion blocks the induction of a large number of proteins known to comprise the SASP (Figure 2b and Figure S4a,c, Supporting Information), suggesting that BCAA transport is a requirement for SASP generation during senescence induction. Similarly, ectopic BCAT1 expression also reduced the expression of the majority of SASP factors without affecting the levels of p16, Lamin B1, or SA‐β‐Gal (Figure 2c,e, and Figures S3b,d,f and S4b,d, Supporting Information). To further explore the functional role of BCAAs in cellular senescence, we utilized the SLC6A15 inhibitor loratadine.^[^ ^61^ ^]^ Immunoblots and RT‐qPCR both showed that loratadine treatment phenocopies SLC6A15 knockdown in senescent cells: upon inducing senescence with either oncogene activation or DNA damage, pharmacological SLC6A15 inhibition blocks the upregulation of several major SASP factors including IL6, IL8, Interleukin 1 alpha (IL1A), and Interleukin 1 beta (IL1B) in a dose‐dependent manner, without significantly altering the levels of p16, p21 (Cyclin Dependent Kinase Inhibitor 1A), or Lamin B1 (Figure S5a–f, Supporting Information). These data suggest that either suppressing BCAA transport (via SLC6A15 inhibition) or activating BCAA catabolism (via BCAT1 expression) both suppress the SASP program in various senescent cell models.

In senescent cells, the cell‐cycle arrest phenotype is induced by the p53‐p21‐phospho‐retinoblastoma protein (RB) and p16‐pRB signaling pathways, while SASP factor expression is thought to be mediated by the Nuclear factor‐κB and CCAAT/enhancer‐binding protein beta transcription factors.^[^ ^62^ ^]^ Despite the existence of crosstalk between these mechanisms controlling cell cycle arrest and the SASP, our results suggest that their regulation can be uncoupled due to the involvement of these distinct signaling pathways, a notion that is consistent with findings from several other recent studies.^[^ ^51^, ^63^, ^64^, ^65^, ^66^, ^67^ ^]^

### 2.3 BCAA Regulators Drive SASP Factor Production by Activating mTORC1 Signaling

**Figure 3:**
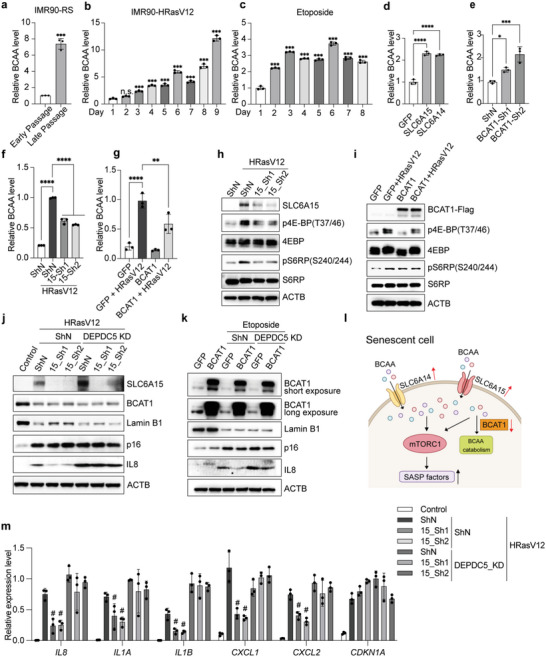
BCAA accumulation drives the SASP by activating mTORC1 signaling. BCAA metabolite assay showing that intracellular BCAA levels increase in a) replicative, b) oncogene‐induced, and c) DNA damage‐induced senescence. Relative BCAA levels were quantified by normalizing to cell number and then to the proliferative control for each condition. d) SLC6A14 or SLC6A15 overexpression increases intracellular BCAA levels in HEK293T cells. e) BCAT1 knockdown in HEK293T cells increases intracellular BCAA levels. a–e) *n* = 3, mean ± SD, a) two‐tailed Student's t‐test or b–e) one‐way ANOVA test with Dunnett's multiple comparisons. *P < 0.05, ***P < 0.001, ****P < 0.0001, ns, not significant. f) SLC6A15 knockdown impairs BCAA accumulation in senescent IMR90 cells. g) BCAT1 rescue impairs BCAA accumulation in senescent IMR90 cells. f,g) *n* = 3, mean ± SD, two‐way ANOVA test with Tukey's multiple comparisons. **P < 0.01, ****P < 0.0001. h) SLC6A15 knockdown or i) BCAT1 overexpression inhibits mTORC1 activation. j,k,m) mTORC1 activation via DEPDC5 knockdown rescues SASP production in j,m) SLC6A15‐depleted or k) BCAT‐overexpressing senescent cells. m) *n* = 3, mean ± SD, two‐way ANOVA test with Tukey's multiple comparisons. The symbol “#” indicates statistical significance (*P* < 0.05) compared to the HRasV12‐ShN‐ShN group. l) Schematic of BCAA‐dependent SASP induction. Increase amino acid transport through SLC6A14 or SLC6A15 together with reduced BCAT1‐mediated catabolism leads to BCAA accumulation in senescent cells. BCAA buildup then activates mTORC1 signaling to promote SASP factor production. RS: replicative senescence; p4EBP(T37/46): eukaryotic translation initiation factor 4E‐binding protein 1 phosphorylated at threonine 37 and/or threonine 46; 4EBP: eukaryotic translation initiation factor 4E‐binding protein 1; pS6RP(S240/244): S6 ribosomal protein phosphorylated at serine 240 and serine 244; S6RP: S6 ribosomal protein; DEPDC5: DEP domain–containing 5; IL1A: interleukin 1 alpha; IL1B: interleukin 1 beta; CXCL1: chemokine (C‐X‐C motif) ligand 1; CXCL2: chemokine (C‐X‐C motif) ligand 2.

To determine the molecular mechanism by which BCAAs mediate SASP production, we first analyzed BCAA levels in senescent cells using an enzymatic assay. As expected, we observed significantly elevated intracellular BCAA levels in all tested senescence models as compared with proliferating cells (**Figure** **3**a–c). Moreover, overexpression of SLC6A14 or SLC6A15 in HEK293T cells resulted in increased BCAA level (Figure 3d), while BCAT1 downregulation produced a similar effect (Figure 3e). Conversely, suppressing SLC6A15 upregulation or restoring BCAT1 expression in senescent cells significantly reduced BCAA accumulation (Figure 3f,g). Together, these results validate that elevated BCAA uptake through SLC6A transporters and reduced BCAT1 catabolism both function to increase BCAA levels in senescent cells.

We next investigated whether increased cellular BCAAs affect SASP establishment by culturing cells in BCAA‐restricted media. This revealed that reducing BCAA levels to 25% of the standard concentration did not affect cell growth (Figure S6a, Supporting Information), but significantly inhibited the expression of multiple important SASP factors (Figure S6b–d, Supporting Information); this is consistent with phenotypes observed upon manipulating BCAA regulators in senescent cells. To further explore metabolic changes in senescent cells, we performed high‐performance liquid chromatography‐mass spectrometry (HPLC‐MS)‐based metabolite profiling. Interestingly, this revealed that, in addition to BCAAs, other essential amino acids are also elevated in two senescent cell models (Figure S7a, Supporting Information); this might be due to broad‐spectrum transport by SLC6A14/15, which mediates the uptake of other essential amino acids in addition to having a high affinity for BCAAs.^[^ ^52^, ^58^, ^59^ ^]^ Consistent with this, transient overexpression of SLC6A14 or SLC6A15 increased intracellular levels of essential amino acids (Figure S7c, Supporting Information), whereas sustained SLC6A15 knockdown significantly reduced nearly all essential amino acids (Figure S7b, Supporting Information). To assess the impact of specific amino acids on SASP establishment, we individually increased the levels of essential amino acids known to be transported by SLC6A14/15, as well as two non‐essential amino acids. This revealed that, among the eight tested amino acids, increasing the levels of BCAAs and tryptophan promotes heightened expression of the SASP factors IL6 and IL8 during senescence (Figure S7d, Supporting Information). Based on these amino acid restriction and supplementation results, we conclude that BCAA accumulation via increased BCAA transport and reduced BCAA catabolism is both necessary for full SASP factor production and also sufficient to induce the expression of key SASP proteins in senescent cells.

BCAAs, particularly leucine, are potent activators of mTORC1,^[^ ^2^ ^]^ and recent studies have shown that mTORC1 signaling drives the SASP program in senescent cells.^[^ ^64^, ^66^, ^68^, ^69^ ^]^ This occurs both through regulating the translation of mitogen‐activated protein kinase‐activated protein kinase 2 (MAPKAPK2) and IL1A to increase the mRNA levels of SASP factors, and also through directly promoting the translation of those mRNAs.^[^ ^64^, ^66^ ^]^ We therefore investigated whether changes in SLC6A15 and BCAT1 influence the mTORC1 signaling pathway. Indeed, SLC6A15 knockdown (Figure 3h and Figure S8a, Supporting Information) or forced BCAT1 expression (Figure 3i and Figure S8b, Supporting Information) inhibited mTORC1 signaling activation during the onset of senescence, suggesting that BCAA buildup is the upstream activator of this pathway. To further confirm that mTORC1 functions downstream of SLC6A15 and BCAT1, we knocked down DEP domain‐containing 5 (DEPDC5) protein, an inhibitory component of the mTORC1 pathway, to reactivate mTORC1.^[^ ^70^ ^]^ This revealed that, even after depleting SLC6A15 or overexpressing BCAT1, rescuing mTORC1 signaling restores the expression of multiple prominent SASP factors (Figure 3j,k,m and Figure S8c,d, Supporting Information). Together, these data suggest that elevated SLC6A15 and reduced BCAT1 expression in senescent cells control SASP establishment via the BCAA‐mTORC1 signaling pathway (Figure 3l).

### 2.4 Upregulating BCAA Transporters Induces Age‐Related Phenotypes in *Drosophila melanogaster*

SASP factors have been shown to have deleterious effects by promoting paracrine senescence, which contributes to aging and inflammatory diseases as the immune system becomes less effective in removing senescent cells.^[^ ^71^, ^72^, ^73^, ^74^ ^]^ To investigate the effects of alterations in BCAA metabolism on the aging process, we turned to *Drosophila melanogaster*, which has a shorter lifespan and shares conserved signaling pathways in cellular senescence and aging with mammals.^[^ ^75^, ^76^, ^77^ ^]^ Furthermore, recent *Drosophila* studies demonstrated that BCAA‐restricted diet extends lifespan,^[^ ^78^, ^79^ ^]^ highlighting the role of BCAA metabolism during physiological aging in *Drosophila*.

**Figure 4:**
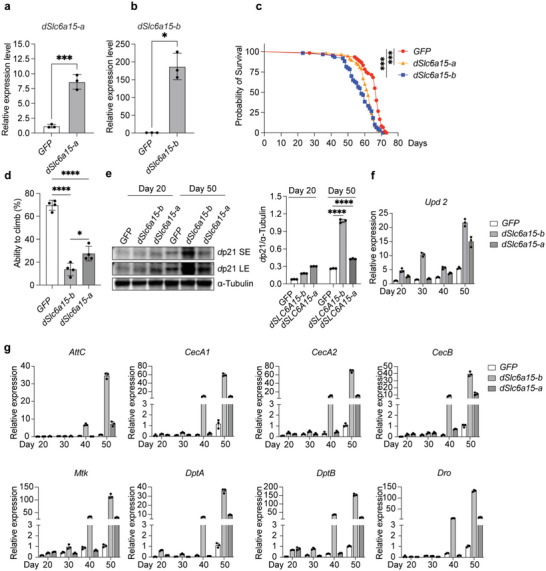
BCAA metabolism regulates cellular senescence and age‐related phenotypes in *Drosophila*. a) Overexpression of dSlc6a15‐a or b) dSlc6a15‐b in transgenic fruit flies using the actin‐Gal4/UAS system. *n* = 3, mean ± SD, two‐tailed Student's t‐test. *P < 0.05, **P < 0.01. c) dSlc6a15‐a or dSlc6a15‐b overexpression shortens fly lifespans and d) impairs climbing ability in 4‐week‐old female flies. c) *n* = 70–75, log‐rank test, ***P < 0.001. d) *n* = 30–40, One‐way ANOVA, *P < 0.05, **** *P* < 0.0001. e–g) dSlc6a15‐a or dSlc6a15‐b overexpression induces expression of e) *Drosophila* dacapo (dp21), f) Upd2, and g) multiple antimicrobial peptides. *n* = 3, mean ± SD. SE: short exposure; LE: long exposure; Upd2: Unpaired 2; Attc: Attacin‐C; CecA1: Cecropin A1; CecA2: Cecropin A2; CecB: Cecropin B; Mtk: Metchnikowin; DptA: Diptericin A; DptB: Diptericin B; Dro: Drosocin.

Based on protein similarity,^[^ ^80^ ^]^ while we could not find an ortholog of human SLC6A14 in fruit flies, we identified two possible orthologs of human SLC6A15: *CG43066* (43.8% identity) and *CG10804* (41.2% identity). Based on the Alliance algorithm,^[^ ^81^ ^]^ the top predicted ortholog for *CG43066* (hereafter termed *dSlc6a15‐a*) is indeed SLC6A15. For *CG10804* (hereafter *dSlc6a15‐b*), the predicted orthologs are SLC6A15, SLC6A17, and SLC6A18. As the functions of these two proteins have not been characterized in flies, and the three mammalian SLC6 family transporters are similar and likely redundant,^[^ ^82^ ^]^ we tested the effects of manipulating the expression of *dSlc6a15‐a* and *dSlc6a15‐b* on fly aging. Specifically, we mimicked the increased BCAA transporter expression observed in senescent mammalian cells by inducing overexpression of *dSlc6a15‐a* or *dSlc6a15‐b* in adult flies using the *actin*‐transcription activator protein Gal4/ Upstream Activation Sequence (Gal4/UAS) system (**Figure** **4**a,b). We used female fruit flies in these experiments, based on our preliminary findings that they were more responsive to the BCAA‐restricted diet and showed a greater lifespan extension compared to male flies, which is consistent with a previous study.^[^ ^79^ ^]^ Strikingly, overexpression of either transporter significantly shortened fly lifespan (Figure 4c) and led to age‐related decline in climbing ability (Figure 4d).^[^ ^83^ ^]^ Additionally, overexpression of *dSlc6a15‐a* or *dSlc6a15‐b* induced cellular senescent hallmarks, including upregulation of the cyclin‐dependent kinase inhibitor *dacapo* (*d*p21) (Figure 4e), and increased expression of the inflammatory cytokine unpaired 2 (*Upd2*) (Figure 4f) in elderly flies. These results suggest that hyper‐expression of BCAA transporters is sufficient to accelerate fly aging, possibly through increased cellular senescence and a pro‐inflammatory SASP.

Previous studies have shown that the expression of antimicrobial peptides is upregulated in aged fruit flies, possibly due to Janus kinase/signal transducers and activators of transcription signaling activation by SASP factors as well as increased exposure to environmental bacteria during aging.^[^ ^84^, ^85^, ^86^, ^87^ ^]^ Strikingly, we observed massive increases in antimicrobial peptide expression in elderly transgenic flies overexpressing BCAA transporters as compared with GFP‐overexpression control flies (Figure 4g). While the detailed mechanism of how this pathway contribute to fly aging needs to be further resolved in future studies, our findings support the notion that increasing BCAA transport shortens fly lifespan and induces senescent as well as aging‐related phenotypes at the organismal level.

### 2.5 Blocking BCAA Accumulation Attenuates the Senescence‐Associated Inflammatory Response in Mouse Liver

**Figure 5:**
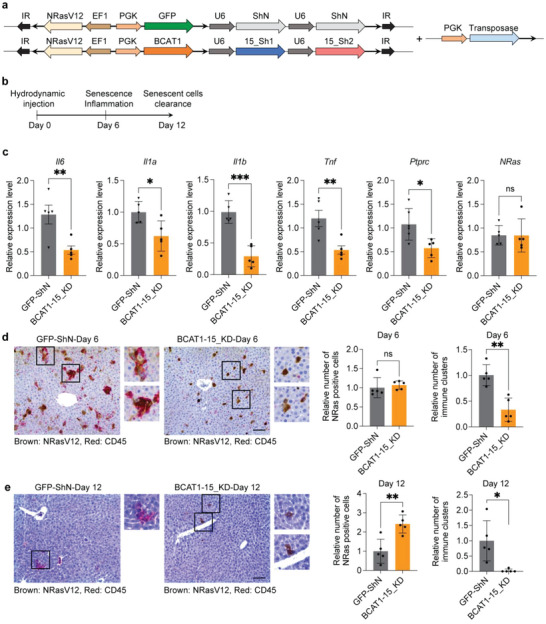
BCAA inhibition abrogates SASP factor production and senescent cell immune clearance in mice. Schematic illustrations of a) sleeping‐beauty transposon plasmids and b) experimental design. c) RT‐qPCR analysis showing that BCAT1 overexpression and SLC6A15 knockdown reduce SASP factor expression and immune cell markers on day 6. Representative images and quantification of liver tissue immunohistochemical staining for NRasV12 (brown) and CD45 (red) expression on d) days 6 and e) 12 in GFP‐ShN control and BCAT1‐15KD groups, as indicated. c–e) *n* = 5, mean ± SD, two‐tailed Student's t‐test. *P < 0.05, **P < 0.01, ***P < 0.001, ns, not significant. Scale bar = 100 µm. IR: inverted repeat; NRasV12: NRas proto‐oncogene with G12V mutation; EF1: elongation factor‐1 promoter; PGK: phosphoglycerate kinase promoter; U6: U6 promoter; Il6: interleukin 6; Il1a: interleukin 1 alpha; Il1b: interleukin 1 beta; Tnf: tumor necrosis factor; Ptprc: protein tyrosine phosphatase receptor type c; CD45: protein tyrosine phosphatase receptor type C.

To investigate the impact of BCAA regulators on the senescence‐associated inflammatory response in the mammalian system, we utilized an established oncogene‐induced senescence model to study the immune‐mediated clearance of senescent mouse hepatocytes.^[^ ^88^ ^]^ This sleeping‐beauty transposon system, which is introduced into mouse liver via hydrodynamic tail vein injection, enables the simultaneous manipulation of multiple genes; here, we knocked down SLC6A15 and overexpressed BCAT1 to reduce intracellular BCAA levels, while also inducing senescence via NRasV12 (NRas proto‐oncogene with G12V mutation) overexpression (**Figure** **5**a,b). Samples were collected on days 6 and 12 to evaluate the effects of BCAA metabolism on SASP gene expression and senescent cell immune clearance, respectively (Figure 5b).

As expected, NRasV12 activation (as indicated in brown) (Figure 5d) induced senescence in hepatocytes, resulting in the rapid production of proinflammatory factors: day 6 analysis revealed high expression of SASP components in the control group (GFP‐ShN) (Figure 5c), along with a large number of protein tyrosine phosphatase receptor type C (CD45)‐positive immune cell clusters (as indicated in red) (Figure 5d). Conversely, in the animals with depleted SLC6A15 and overexpressed BCAT1 (BCAT1‐SLC6A15_KD), we observed significantly attenuated expression of canonical SASP factors (Figure 5c), with few immune cell clusters present (as indicated in red) (Figure 5d). These results suggest that, if BCAA accumulation is impaired, senescence can still be induced by oncogene activation, but SASP factor production is blocked such that senescent cells are not efficiently cleared by the immune system. Consistent with this, on day 12, significantly fewer NRasV12‐positive cells (as indicated in brown) were observed in the control group (GFP‐ShN) as compared with the experimental group (BCAT1‐SLC6A15_KD) (Figure 5e). Collectively, these findings support the notion that elevated BCAA levels are required for the senescence‐associated inflammatory response and immune clearance of senescent cells in vivo.

To explore whether BCAA metabolism is also involved in the mammalian aging process, we examined the relationship between expression of SLC6A14/15 and BCAT1/2 with a panel of established SASP transcripts across various human datasets obtained from the Genotype‐Tissue Expression (GTEx) portal.^[^ ^89^ ^]^ This analysis revealed a positive correlation between SLC6A14 and SLC6A15 and the SASP signature score, whereas BCAT1 and BCAT2 levels showed a negative correlation with the SASP signature score across multiple aged human tissues, including lung, pancreas, and stomach (Figure S9a–e, Supporting Information). These findings indicate that elevated BCAA transport and reduced BCAA degradation are associated with enhanced pro‐inflammatory cytokine expression during the aging process, suggesting that altered BCAA metabolism could play an important role in age‐related chronic inflammation in humans.

## 3 Discussion

While previous studies have reported that circulating BCAA levels are altered during physiological aging and age‐related diseases,^[^ ^5^, ^90^, ^91^, ^92^, ^93^, ^94^ ^]^ including obesity, type 2 diabetes, cardiovascular disease, and Alzheimer's disease, the metabolic pathways underlying altered BCAA abundance in these settings have remained largely obscure. Moreover, while BCAAs are a popular dietary supplement with purported benefits for physical exercise,^[^ ^4^ ^]^ liver disease,^[^ ^95^ ^]^ and brain function,^[^ ^96^ ^]^ their roles in aging and inflammation‐related diseases remain controversial. This controversy is due to various factors,^[^ ^5^ ^]^ including an unclear causality between blood BCAA levels and disease onset or progression, poor correlation between dietary BCAA intake and blood availability, variability in BCAA requirements among organs, and the fact that BCAAs in the blood do not always reflect those in cells. Therefore, it is important to understand the intrinsic regulation of BCAA metabolism in these contexts in order to develop more precise nutritional interventions or combination therapies. Here, we discovered that senescent cells reprogram BCAA metabolism by increasing BCAA uptake and decreasing catabolism; together, these intrinsic metabolic alterations may contribute to the changes in blood BCAA levels observed in age‐ and inflammation‐related diseases. This has important implications for these age‐related disorders, as senescence plays a crucial role in them.^[^ ^21^, ^22^, ^23^, ^24^, ^25^ ^]^ SASP is akin to a double‐edged sword. On the one hand, SASP factors play a beneficial role by recruiting immune cells to eliminate senescent cells triggered by oncogene expression, DNA damage, or other stressors, potentially preventing oncogenesis in young and healthy populations.^[^ ^10^, ^11^, ^12^, ^13^, ^14^ ^]^ However, as the immune system's efficacy diminishes with age and stress stimuli accumulate, there is a decreased efficiency in the clearance of senescent cells. This results in prolonged SASP activity, which can culminate in detrimental inflammation and expedite disease progression.^[^ ^10^, ^11^, ^12^, ^13^, ^14^ ^]^ It is also worth noting that BCAA supplementation should be used with caution in the elderly and those with inflammatory diseases, as intracellular BCAA accumulation is shown here to promote a senescence‐associated‐inflammatory response.

In addition to their impact on the SASP during aging, BCAAs also influence other aging‐related signaling pathways. One of the hallmarks of aging is diminished mitochondrial functionality.^[^ ^97^ ^]^ BCAAs, along with related metabolites, have been shown to modulate mitochondrial biogenesis and functions.^[^ ^97^ ^]^ The underlying mechanisms for these modulations are evidently associated with the mTORC1 and Sirtuin 1 pathways and the regulation of TCA cycle enzyme activities.^[^ ^97^ ^]^ Furthermore, BCAA restriction has shown promise in improving insulin resistance and reducing body fat by modulating the energy‐regulating hormone fibroblast growth factor 21 (FGF21) to enhance metabolic health.^[^ ^98^ ^]^

BCAAs are often studied collectively due to their shared structural and metabolic features. However, a closer examination reveals both overlapping and distinct roles for individual BCAAs across various age‐related diseases. For instance, dietary restriction of all or each of the three BCAAs results in improved metabolic health, primarily through modulation of FGF21.^[^ ^98^, ^99^, ^100^ ^]^ Notably, among BCAAs, isoleucine restriction stands out as the most potent.^[^ ^98^, ^99^, ^100^ ^]^ In atherosclerosis, leucine uniquely demonstrates anti‐atherogenic properties through reducing macrophage triglyceride content to decrease macrophage foam cell formation, a critical step in the development of atherosclerosis.^[^ ^101^ ^]^ In Alzheimer's disease research, a salient decline in circulating valine levels has been consistently documented, and this appears intricately linked to cognitive degeneration.^[^ ^90^ ^]^ While the exact mechanisms underlying this association remain to be elucidated, it is noteworthy that dietary BCAA restriction manifests favorable outcomes, whereas BCAA supplementation appears deleterious in Alzheimer's disease mouse models.^[^ ^9^ ^]^ Given the intricacies of these findings, further research is essential to unravel both the collective and distinct roles played by each BCAA in the context of age‐related disorders.

Although they did not report BCAAs’ specific role in inducing the SASP, previous studies have shown these amino acids’ involvement in various age‐ and inflammation‐related diseases. For example, deletion of SLC6A15 has been shown to improve motor performance in aged mice^[^ ^102^ ^]^ and its expression has been found to positively correlate with neuropathic and inflammatory pain.^[^ ^103^ ^]^ Similarly, depletion of SLC6A19, another member of the SLC6 family that is enriched in kidney, leads to reduced senescent markers and inflammation in aristolochic acid‐induced kidney injury.^[^ ^104^ ^]^ Additionally, a loss in BCAT1 has been observed in aged mouse brains and Alzheimer's disease models,^[^ ^105^ ^]^ while its knockdown has been reported to cause Parkinson's disease‐like progressive motor deficits and neurodegeneration with age in *Caenorhabditis elegans*.^[^ ^106^ ^]^ These findings support our discovery that enhanced BCAA transporter expression or reduced BCAA catabolism promotes senescence and inflammation. In addition, we observed that the expression of BCAA regulators correlates with inflammation signatures in multiple aged human tissues. Therefore, small molecules that enhance BCAA catabolism^[^ ^107^ ^]^ or inhibit BCAA transporters^[^ ^61^, ^108^ ^]^ should be investigated for their potential efficacy in treating various age‐related and inflammatory conditions.

While our study provides new insights into the role of BCAA regulators in the SASP and their impact on senescence‐associated inflammation, it is important to acknowledge its limitations. BCAA regulators may have additional roles in the aging process by modulating various signaling pathways.^[^ ^109^ ^]^ For example, recent research revealed that BCAT regulates isoleucine abundance in neurons to influence the feeling of hunger and affect the lifespan of fruit flies.^[^ ^79^ ^]^ Moreover, SLC6A15 knockdown was shown to impact appetite and weight gain in mice,^[^ ^110^ ^]^ which may affect their life span and metabolic health in the long term. These findings highlight the need for further research to explore the complex roles of BCAA regulators in organismal aging. These complexities notwithstanding, our findings uncover an essential role for intrinsic metabolic reprogramming, leading to BCAA accumulation, in establishing the pro‐inflammatory SASP upon senescence induction, with important implications for human aging and inflammation‐related diseases.

## 4 Experimental Section

### Cell Culture

Human IMR90 lung diploid fibroblasts and HEK293T cells were purchased from Duke University's Cell Culture Facility (originally from American Type Culture Collection (ATCC). Normal human bronchial epithelial (NHBE) cells derived from healthy donors were purchased from LONZA. IMR90 cells were cultured in Eagle's minimum essential medium (MEM) supplemented with 10% fetal bovine serum (FBS), 1 x MEM non‐essential amino acids, 1 × 10^‐3^ m pyruvate, and 100 U mL^‐1^ penicillin‐streptomycin. HEK293T cells were cultured in Dulbecco's modified Eagle's medium (DMEM) supplemented 10% fetal bovine serum (FBS) and 100 U mL^‐1^ penicillin‐streptomycin. NHBE cells were cultured in basal epithelial growth medium (BEGM). All cells were maintained at 37 °C incubator with 5% CO_2_.

### Plasmids

ShRNA vectors targeting SLC6A15 and DEPDC5 were purchased from Sigma‐Aldrich. Coding sequences of human SLC6A14, SLC6A15, and BCAT1 were subcloned into pSIN‐lentiviral vector using Gibson Assembly Master Mix kit (E2611S, NEB). The CDS sequences of human HRasV12 were inserted into the lentiviral vector pCDH‐TetOne‐MCS‐EF1‐Puro.^[^ ^49^ ^]^ PAX2 (Addgene, 12 260) and VSV‐G (Addgene, 12 259) were gifts from D. Trono. The shRNA targeting sequences and the primers used for cloning were provided in Table S1 (Supporting Information).

### Stable Cell Lines

HEK293T cells were seeded and transfected with lentiviral plasmids together with PAX2 and VSV‐G using Lipofectamine 2000 (Thermo Fisher Scientific) according to the manufacturer's protocol. Virus‐containing media were collected 48 hours post‐transfection and filtered using 0.45 × 10^‐6^ m PVDF filters to remove cells. These media were used to infect target cells for 12 hours. Two days later, infected cells were subjected to puromycin treatment for 2 to 4 d to establish stable lines.

### RT‐qPCR

Total RNA was extracted using RNeasy Mini Kit (QIAGEN). Equal amounts of RNA were reverse transcribed using the iScript gDNA Clear cDNA Synthesis Kit (Bio‐Rad). Synthesized cDNA was analyzed by quantitative PCR with SYBR Green (Roche). Primer sequences for all tested genes are provided in Table S2 (Supporting Information).

### Western Blotting and Antibodies

Cell pellets were lysed using RIPA buffer with protease and phosphatase inhibitor cocktails (Thermo Fisher Scientific) and 1% SDS. The resulting cell lysates were boiled for 10 min, and protein concentrations were determined by BCA Protein Assay (Thermo Fisher Scientific). Volumes were adjusted to ensure that all samples had equivalent protein concentrations, and samples were boiled in 1x loading and reducing buffer (Thermo Fisher Scientific). Samples were loaded onto denaturing SDS polyacrylamide gels and separated proteins were transferred onto polyvinylidene difluoride membranes. Membranes were blocked with 5% BSA in TBS buffer and then incubated with primary antibodies overnight. Corresponding HRP‐conjugated secondary antibodies and chemiluminescent horseradish peroxidase substrate (Thermo Fisher Scientific) were used for signal detection.

Antibodies used in this study are as follows: Beta Actin (Proteintech, 66009‐1‐Ig, 1:5000), p16 (BD, 551154, 1:1000), p21 (Cell Signaling, 2947, 1:1000), p21 (Mouse Preferred) (Cell Signaling, 64016, 1:1000), IL‐6 (Cell Signaling, 12153, 1:1000), IL‐8 (Abcam, ab18672 1:1000), Ras (G12V Mutant Specific) (Cell Signaling, 14412, 1:1000), SLC6A14 (Sigma‐Aldrich, HPA003193, 1:1000), SLC6A15 (Abcam, ab191192, 1:1000), BCAT1 (Proteintech, 13640‐1‐AP, 1:1000), BCAT2 (Proteintech, 16417‐1‐AP, 1:1000), Lamin B1 (Proteintech, 12987‐1‐AP, 1:1000), Phospho‐p70 S6 Kinase (Cell Signaling, 9205, 1:1000), p70 S6 Kinase (Cell Signaling, 2708, 1:1000), Phospho‐S6 Ribosomal Protein (Ser240/244) (Cell Signaling, 5364, 1:1000), S6 Ribosomal Protein (Cell Signaling, 2317, 1:1000), Phospho‐4E‐BP1 (Thr37/46) (Cell Signaling, 2855, 1:1000), 4E‐BP1 (Cell Signaling, 9644, 1:1000), Dacapo (*d*p21)(Developmental Studies Hybridoma Bank, NP1, AB_10805540), Anti‐mouse HRP (Cell Signaling, 7076, 1:5000), Anti‐Rabbit HRP (Thermo Fisher, G‐21234, 1:5000).

### IHC Staining

Mouse liver tissues were collected and fixed in 10% formalin overnight and then embedded in paraffin and sectioned into 8 µm slices. Slides were deparaffinized and rehydrated in graded ethanol followed by antigen retrieval in Decloaker buffer (Biocare Medical). Next, slides were treated with 3% H_2_O_2_ to block endogenous peroxidase activity. 5% BSA was then used to block non‐specific binding. Primary antibodies were added and incubated overnight. After 3 washes with TBST, corresponding secondary antibodies were added and incubated for 1 h. DAB substrate (Dako) and Vibrant Red (Cell Signaling) were used to detect signals, and hematoxylin (Vector Laboratories) was used to stain the nucleus. Images were taken under an Olympus CK40 microscope (Center Valley). The following primary antibodies were used for IHC staining: Ras (G12V Mutant Specific) (Cell Signaling, 14412, 1:100), CD45 (BioLegend, 103101, 1:100), Anti‐Rabbit HRP (Dako, K4003), Anti‐Rat (Invitrogen, A18868, 1:100).

### Induction of Senescence and SA‐β‐Gal Assays

Induction of senescence was performed as described previously.^[^ ^49^ ^]^ Erlotinib‐induced senescence: NHBE were treated with the EGFR inhibitor erlotinib (1 × 10^‐6^ m) for 48 h and then collected at day 7. Oncogene induced senescence: IMR90 cells with Tet‐on HRasV12 were treated with doxycycline (1 µg mL^‐1^) for 9 d. DNA damage‐induced senescence: IMR90 cells were treated with 100 × 10^‐6^ m etoposide for 24 hours to induce DNA damage and then cultured for another 8 d. SA‐β‐Gal assays were conducted using a Senescence‐β‐Galactosidase Staining Kit (Cell Signaling) according to the manufacturer's protocol. In brief, cells were rinsed twice in PBS and fixed with 1 x fixative solution for 5 min. Fixed cells were washed once with PBS and incubated in 1x staining solution with X‐Gal overnight. The staining solution was then removed and switched to 70% glycerol. Photographs were taken using an Olympus CK40 microscope (Center Valley).

### BCAA Analysis

BCAA levels were measured using a BCAA Assay Kit (Abcam) according to the manufacturer's protocol. Briefly, cells were trypsinized and pelleted. BCAA assay buffer was added to lyse the cells followed by full‐speed centrifugation for 10 min to remove cell debris. Reaction reagents were mixed and added to the cell lysates. After 30 min of incubation, the OD was read using a plate reader at 450 nm. BCAA concentrations were calculated using a standard curve.

### Metabolite Measurements

Metabolite extraction has been described previously.^[^ ^111^ ^]^ In brief, equivalent numbers of cells were seeded on six‐well plates and cultured overnight. To extract polar metabolites, 1 mL of prechilled 80% ethanol (HPLC grade) was added to each well after removing the culture medium. The plates were then placed on dry ice and immediately transferred to −80 °C. After incubation for 15 minutes, cells were scraped off with the solvent and transferred to 1.5 mL Eppendorf tube for centrifugation at 20 000 × *g* for 10 min at 4 °C. Next, the resulting supernatant was split into two new tubes and dried in a vacuum concentrator. The dried pellets were reconstituted in 30 µL of sample solvent (water:methanol:acetonitrile, 2:1:1, v/v/v) for analysis by HPLC coupled with Orbitrap Exploris 480 Mass Spectrometer (Thermo). Raw data were analyzed using Compound Discoverer 3.3 software (Thermo).

### Mouse Studies

Eight‐week‐old female C57BL/6 mice were purchased from Charles River Laboratories. The mice were housed in a temperature‐controlled room (72°F) with a humidity range of 30–70% and a 12 h:12 h light:dark cycle. All animal procedures were conducted following institutional and National Institutes of Health guidelines and approved by Duke University's Animal Care and Use Committee (A044‐23‐02). The research involving animals followed all applicable ethical regulations. The induction of senescence in mouse liver using the Sleeping Beauty transposon system was conducted as described in a previous paper.^[^ ^88^ ^]^ In brief, 8‐12 weeks old female mice were injected with 80 µg of NRasV12‐GFP‐ShN (or NRasV12‐BCAT1‐SLC6A15_KD, as indicated) and 40 µg of transposase plasmids diluted in Ringer solution through the tail vein (*n* = 5 per group). The volume of Ringer solution used for injection was 10% of the mouse's body weight, not exceeding 2.5 mL if the mouse weighed over 25 grams. Mice were dissected on days 6 and 12 post‐injection, and livers were collected for further analysis.

### Fruit Fly Studies

Flies were maintained at 22 °C with standard fly food (Archon Scientific). Full‐length *D. melanogaster dSlc6a15‐a* and *dSlc6a15‐b* were cloned into a pPFMW vector by using gateway cloning technology (Thermo) and the resulting plasmids were used to generate transgenic flies (Model System Injections, Duke University). Male transgenic flies were mated with virgin female flies with balancer chromosomes, and offspring with specific phenotypes were selected to generate the stable lines. To study the effects of *dSlc6a15‐a* and *dSlc6a15‐b* on the aging process, stable transgenic flies were crossed with driver lines carrying *actin‐Gal4* and *tubulin‐Gal80^ts^* and raised at 18 °C. Hatching flies were collected within 24 h. Male and female flies were separated and transferred to 30 °C with 1SY food^[^ ^112^ ^]^ after 48 h of mating. For lifespan analysis, female flies were switched into fresh vials daily while dead flies were counted and recorded, *n* = 70–75. For climbing assays, 4 week old female flies were transferred to an empty vial with a line drawn 6 centimeters from the bottom, *n* = 30–40. The vial was tapped gently three times to ensure all flies were on the bottom and kept vertically for about 15 s. Videos were then recorded to analyze the number of flies crossing the 6 cm line within 10 s. The assay was repeated three times and averages were calculated.

### Signaling Pathway Analysis

Microarray expression datasets for proliferative cells and erlotinib‐induced senescent cells were downloaded from GSE100014.^[^ ^49^ ^]^ The differential expression analysis between proliferating and senescent cells was performed by limma (V 3.54.2).^[^ ^113^ ^]^ Gene set enrichment analysis (GSEA) was performed using the R package cluster Profiler (V 4.2.2),^[^ ^114^ ^]^ based on the metric of ‐log(*P* value)*sign(log2 fold change), and the pathway of interests were retrieved from gsea‐msigdb online repository. *P*‐values in the ridgeplot were corrected using the Benjamini‐Hochberg method.

### GSVA and Correlation Analysis

To investigate the correlation between the BCAA regulators and the SASP signature, we performed GSVA^[^ ^115^ ^]^ for the SASP genes. Thirty prominent SASP factors^[^ ^116^ ^]^ (Table S3, Supporting Information) were used to generate a gene set and queried its score in different tissues present in GTEx.^[^ ^89^ ^]^ Human donors with ages greater than 60 were characterized as “aged” and included into the analysis. The correlation test between the GSVA score and the gene of interest was carried out by Pearson correlation.

### Code Availability

Microarray data retrieval, differential expression analysis, GSEA analysis, GSVA analysis, and signature correlation were performed using R package.

### Statistical Analysis

The ImageJ software was used to measure the band intensity for western blotting quantitation. RT‐qPCR and western blot signals were normalized to housekeeping genes/proteins, *n* = 3. BCAA concentrations were normalized by cell numbers, *n* = 3. Further data statistical analyses were conducted using GraphPad Prism software. Significance of results was determined by unpaired two‐tailed student's t‐test, one‐way Analysis of Variance (ANOVA) with Dunnett's multiple comparisons, or Two‐way ANOVA with Tukey's multiple comparisons as indicated in the figure legends. Fly survival was analyzed by Log‐rank (Mantel‐Cox) test, *n* = 70–75. For the climbing assay, data were analyzed by one‐way ANOVA with Dunnett's multiple comparisons, *n* = 30–40. For mouse studies, data were analyzed with a two‐tailed Student's t‐test, *n* = 5. Data were presented as mean ± SD, *P* values below 0.05 were considered statistically significant and are represented in the figures by asterisks, where * denotes *P* < 0.05, ** denotes *P* < 0.01, *** denotes *P* < 0.001, and **** denotes *P* < 0.0001. All of the experiments were repeated with a minimum of two independent repeats with similar results unless otherwise noted.

## Conflict of Interest

The authors declare no conflict of interest.

## Author Contributions

X.F.W., P.B.A., and Y.L. conceived the project. Y.L. performed the majority of the experiments. C.P. contributed to the establishment of in vitro cellular senescence models. T.Y. performed the hydrodynamic tail vein injection. L.W. and Z.Z. contributed to the generation of transgenic flies. X.G. and H.Q. performed HPLC‐MS analysis. E.W. performed the bioinformatic analysis. D.H., L.T., and K.X. contributed to cell culture experiments. Y.W. contributed to the construction of Sleeping Beauty transposon system. Q.J.L. and T.P.Y. contributed to data analysis and experimental design. X.F.W., P.B.A., and Y.L. composed the manuscript. All authors discussed the results and reviewed the manuscript.

## Supporting information

Supporting Information

## Data Availability

The data that support the findings of this study are available in the supplementary material of this article.
