# Supplementary material for: Branched‐Chain Amino Acid Accumulation Fuels the Senescence‐Associated Secretory Phenotype
Source: Adv Sci (Weinh). 2023 Nov 15;11(2):2303489. doi: 10.1002/advs.202303489 (PMC10787106; doi:10.1002/advs.202303489)
Supplement: Supplementary file 1 — Supporting Information [file ADVS-11-2303489-s001.pdf]

ADVANCED SCIENCE
Open Access

Supporting Information

for *Adv. Sci.*, DOI 10.1002/advs.202303489

Branched-Chain Amino Acid Accumulation Fuels the Senescence-Associated Secretory Phenotype

*Yaosi Liang, Christopher Pan, Tao Yin, Lu Wang, Xia Gao, Ergang Wang, Holly Quang, De Huang, Lianmei Tan, Kun Xiang, Yu Wang, Peter B. Alexander, Qi-Jing Li, Tso-Pang Yao, Zhao Zhang and Xiao-Fan Wang**

Supplemental Figure 1. Altered lipid, glucose, and amino acid metabolism in senescent cells.

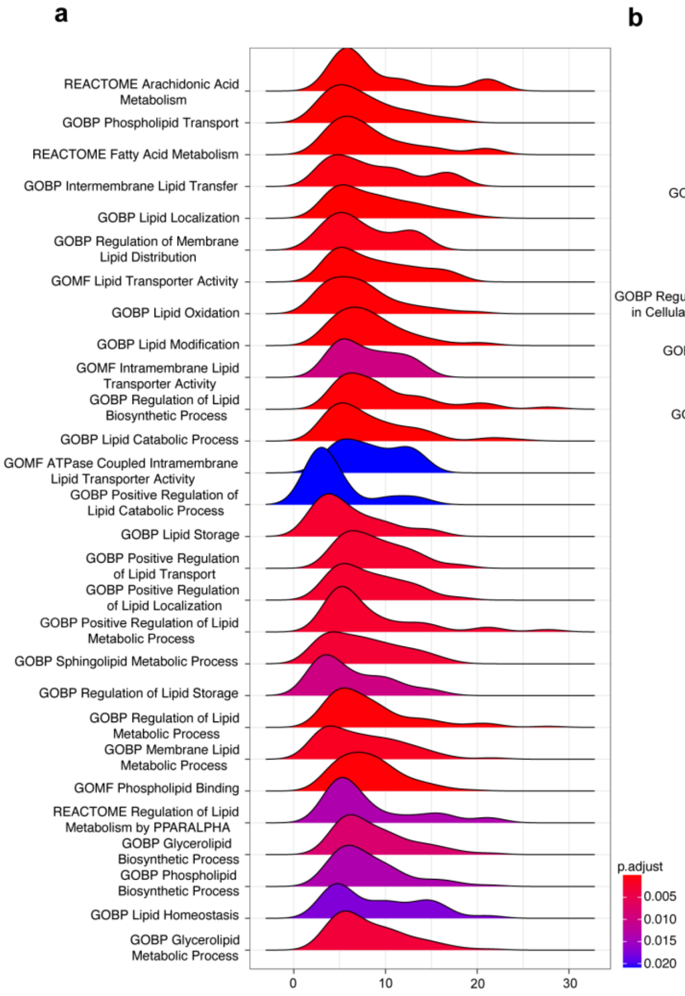

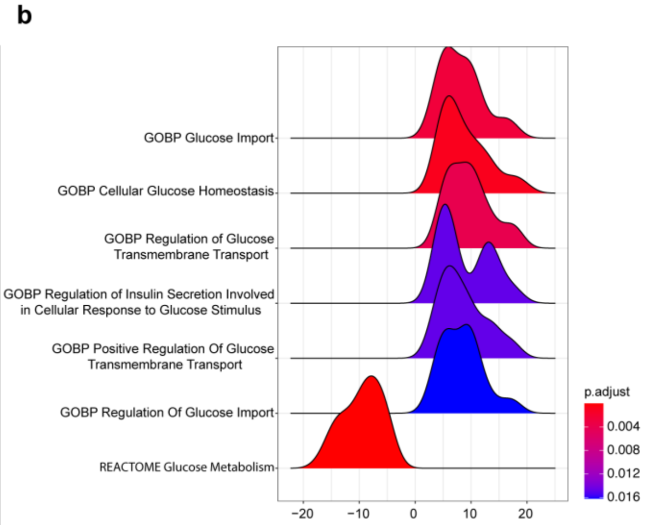

c

Gene	LogFC	Adjusted p-value
SLC6A14	3.11	1.15E-06
CLIC3	3.07	6.75E-08
ASS1	2.80	5.98E-08
OCA2	2.30	5.16E-05
KMO	2.22	6.65E-07

d

Gene	LogFC	Adjusted p-value
BCAT1	-1.69	4.99E-06
CHAC1	-1.69	1.51E-06
DDAH1	-1.48	1.13E-06
PFAS	-1.41	8.68E-06
SLC39A8	-1.36	2.09E-05

Supplemental Figure 1. Altered lipid, glucose, and amino acid metabolism in senescent cells. (a-b) Alterations in lipid- (a) and glucose-related (b) metabolic pathways in senescent cells, as determined by transcriptome profiling of senescent and proliferating NHBE cells. (c) Top 5 upregulated genes in amino acid-related metabolic pathways in senescent NHBE cells compared with proliferative cells. (d) Top 5 downregulated genes in amino acid-related metabolic pathways in senescent NHBE cells compared with proliferative cells.

Supplemental Figure 2. The changes of SLC6A14, SLC6A15 and BCAT1 in senescenct cells.

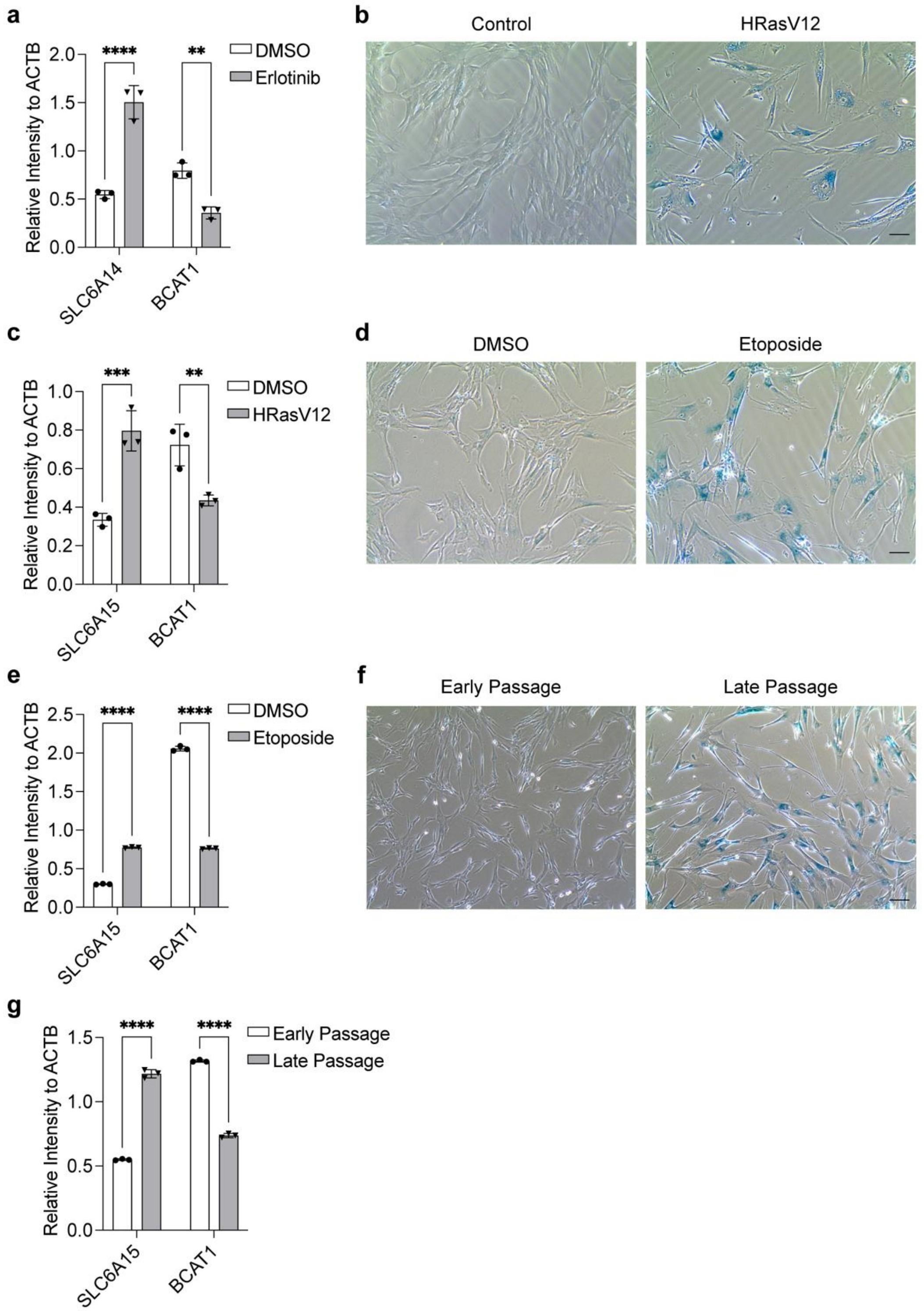

Supplemental Figure 2.The induction of senescence. (a) Quantitation of Figure 1c. (b) Representative SA-β-Gal staining images of oncogene-induced senescence. IMR90 cells with Tet-on HRasV12 were treated with doxycycline (1 μg/mL) for 9 days prior to SA-β-Gal staining.

Scale bar=100 µm. (c) Quantitation of Figure 1e. (d) Representative SA-β-Gal staining images of DNA damage-induced senescence. IMR90 cells were treated with 100 µM etoposide for 24 hours and then cultured for 8 days prior to SA-β-Gal staining. Scale bar=100 µm. (e) Quantitation of Figure 1g. (f) Representative SA-β-Gal staining images of replicative senescence. Early (passage doubling: 38) and late passage (passage doubling: 78) IMR90 cells were stained, as indicated. Scale bar=100 µm. (g) Quantitation of Figure 1i. (a, c, e, g) n=3, mean ± SD, two-way ANOVA test with Tukey's multiple comparisons, *$P < 0.05$, **$P < 0.05$, ***$P < 0.001$, ****$P < 0.0001$.

Supplemental Figure 3. SLC6A15 and BCAT1 regulate SASP factor production.

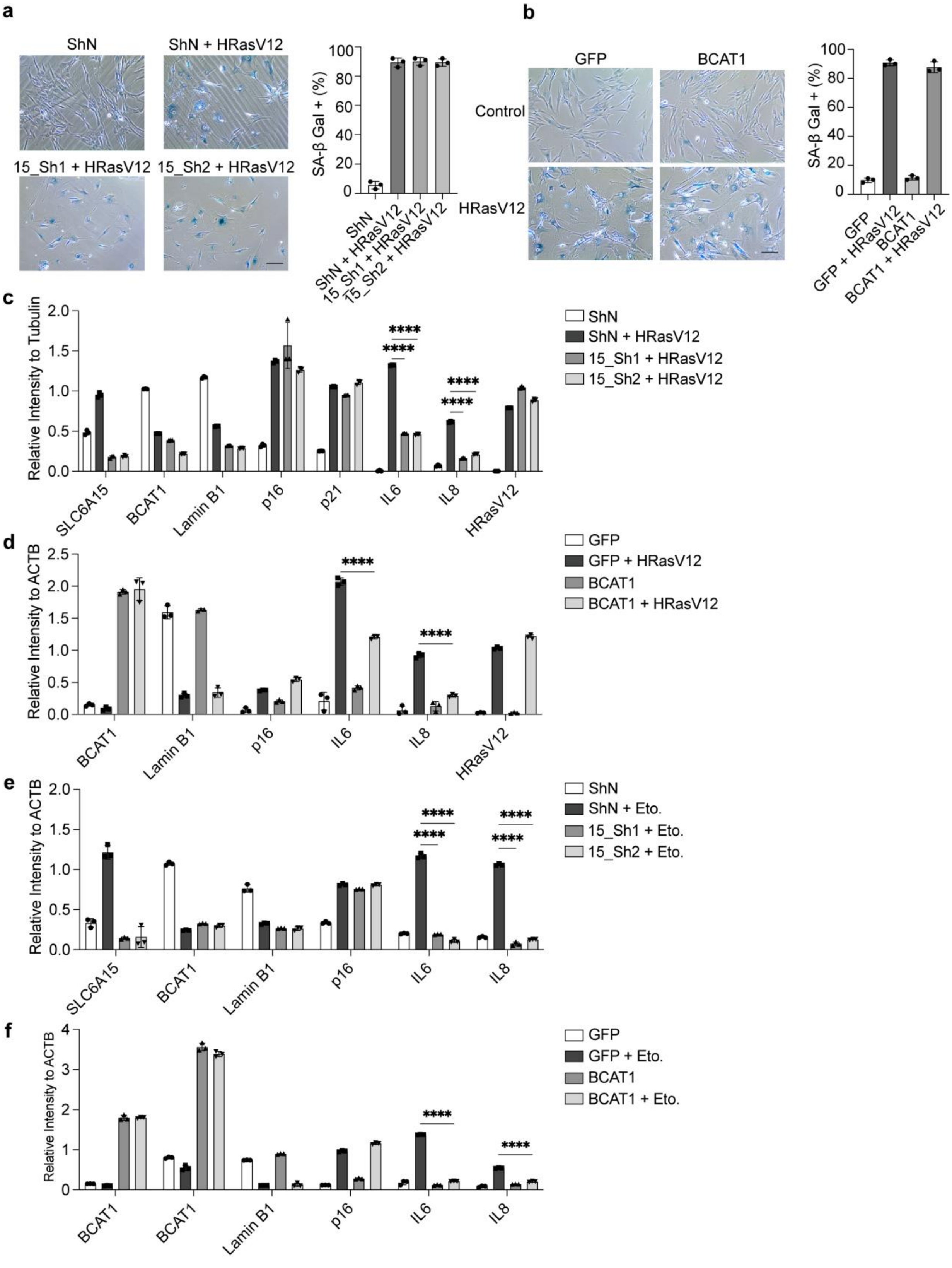

Supplemental Figure 3. SLC6A15 and BCAT1 regulate SASP factor production. (a-b) Representative images and quantitation of SA-β-Gal staining indicate that SLC6A15 knockdown (a) and BCAT1 overexpression (b) do not affect percentages of SA-β-Gal^{+} cells in oncogene-induced senescence. Scale bar=100 µm. (c-f) Quantitation of Figure 2a (c), 2c (d), 2d (e), 2e (f). n=3, mean ± SD, two-way ANOVA test with Tukey's multiple comparisons, ****P < 0.0001.

Supplemental Figure 4. SLC6A15 and BCAT1 regulate the expression of multiple SASP factors.

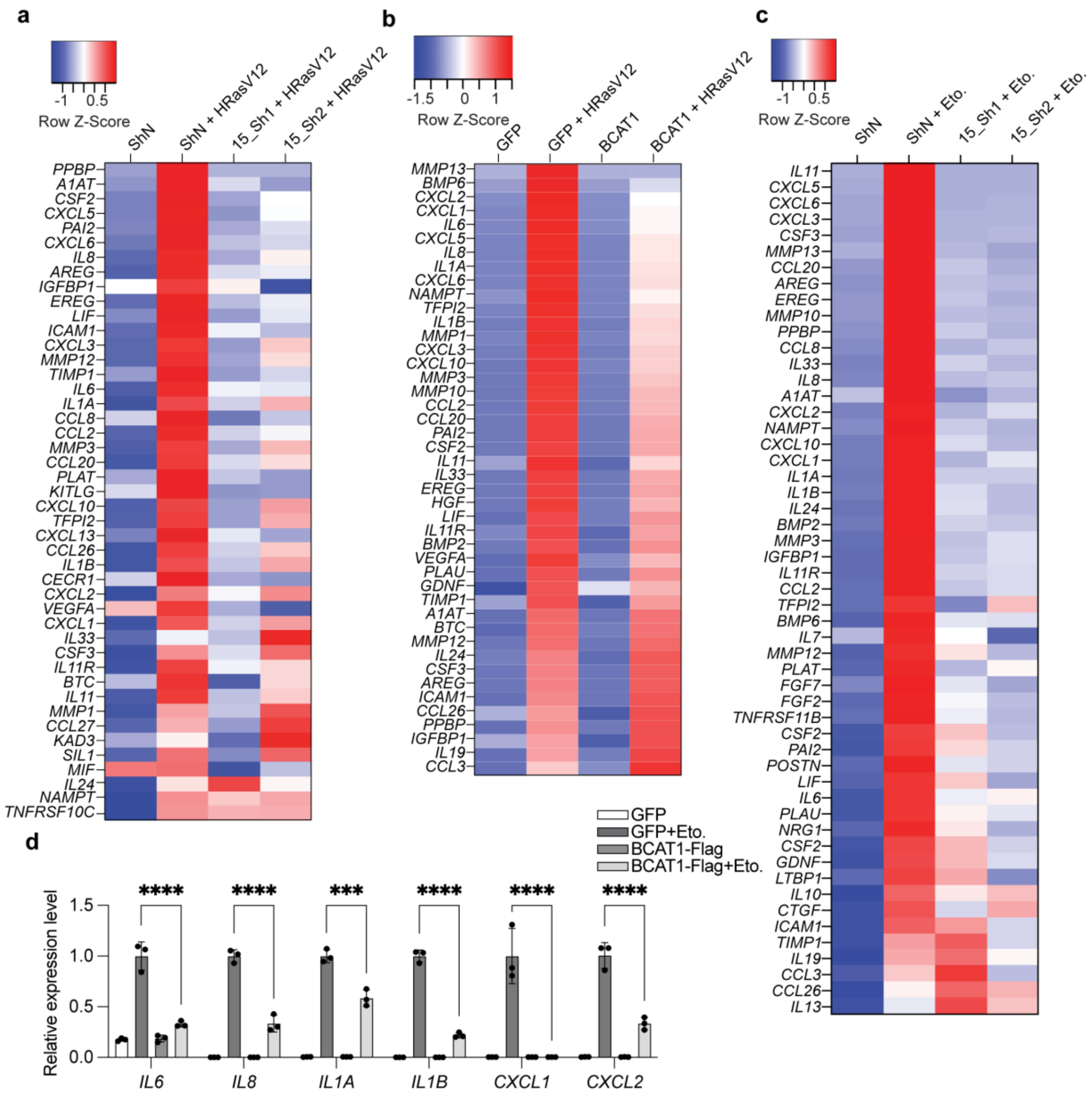

Supplemental Figure 4. SLC6A15 and BCAT1 regulate the expression of multiple SASP factors. (a, b) RT-qPCR analysis showing that SLC6A15 knockdown (a) and BCAT overexpression (b) inhibit the induction of multiple SASP factors in oncogene-induced senescence. (c, d) RT-qPCR analysis showing that SLC6A15 knockdown (c) and BCAT1 overexpression (d) inhibit the induction of multiple SASP factors in DNA damage-induced senescence. (a, b) IMR90 cells with Tet-on HRasV12 were treated with doxycycline (1 μg/mL) for 9 days to induce senescence. (c, d) IMR90 cells were treated with 100 μM etoposide for 24 hours and then cultured for 8 days before collecting samples. Cells were infected with lentivirus to express ShN, SLC6A15 shRNA, GFP, or BCAT1, as indicated. n=3, mean ± SD, two-way ANOVA test with Tukey's multiple comparisons. ***$P < 0.001$, **** $P < 0.0001$.

Supplemental Figure 5. SLC6A15 inhibition blocks the SASP.

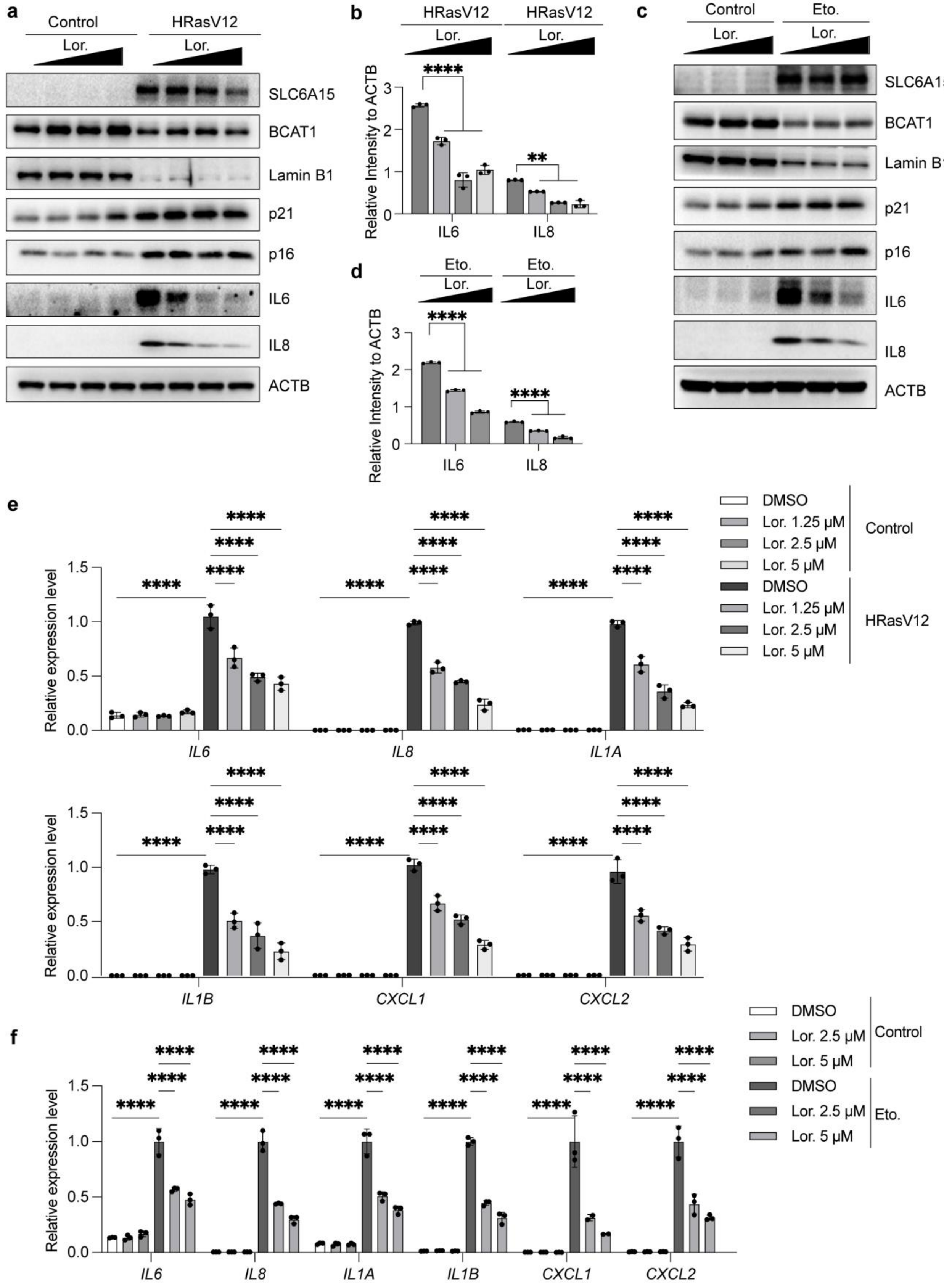

Supplemental Figure 5. SLC6A15 inhibition blocks the SASP. (a) Treatment with the SLC6A15 inhibitor loratadine reduces the expression of multiple SASP factors in oncogene-induced senescence, as determined by immunoblotting (a, quantitated in b) and RT-qPCR analysis(e). IMR90 cells with Tet-on HRasV12 were treated with doxycycline (1 μg/mL) for 9 days to induce senescence. Increasing concentrations of loratadine (0, 1.25, 2.5, or 5 μM) were added to media during senescence induction. (c, d, f) Loratadine treatment reduces the expression of multiple SASP factors in DNA damage-induced senescence as determined by immunoblotting (c, d) and RT-qPCR (f) analysis. IMR90 cells were treated with 100 μM etoposide for 24 hours and then cultured for 8 days before collecting samples. Increasing concentrations of loratadine (0, 2.5, or 5 μM) were added to the media during senescence induction. n=3, mean ± SD, two-way ANOVA test with Tukey's multiple comparisons. **$P < 0.05$, **** $P < 0.0001$. Lor.: loratadine.

Supplemental Figure 6. BCAA deprivation impairs SASP factor expression.

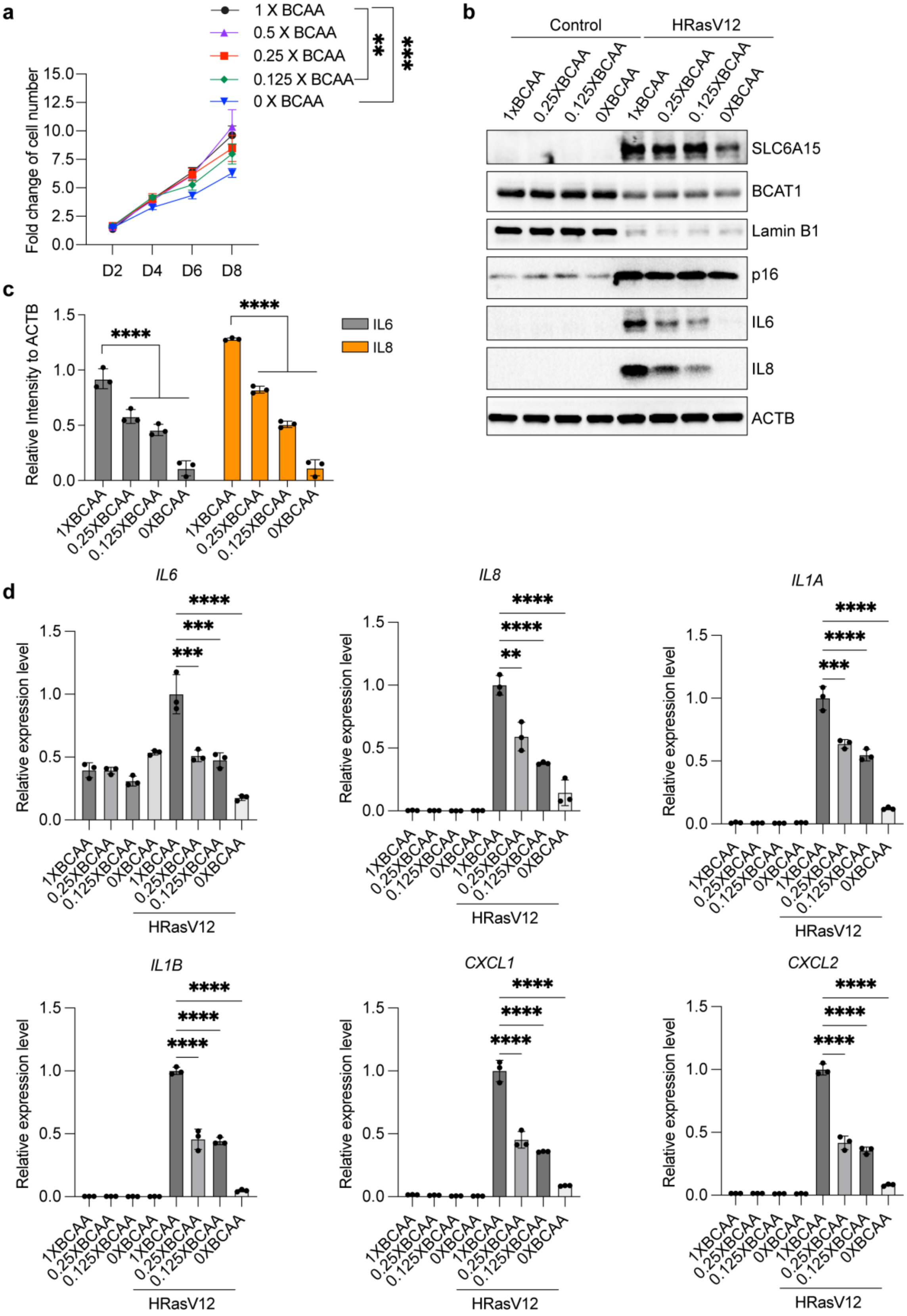

Supplemental Figure 6. BCAA deprivation impairs SASP factor expression. (a) IMR90 cell growth rates in media containing the indicated BCAA concentrations. (b-d) Immunoblots (b, quantitated in c) and RT-qPCR analysis (d) showing that reducing BCAA levels blocks SASP factor induction. IMR90 cells with Tet-on HRasV12 were treated with doxycycline (1 μg/mL) to induce senescence. n=3, mean ± SD, one-way ANOVA test with Dunnett's multiple comparisons test, **P < 0.01, ***P < 0.001, ****P < 0.0001.

Supplemental Figure 7. Changes in essential amino acid levels in senescent cells.

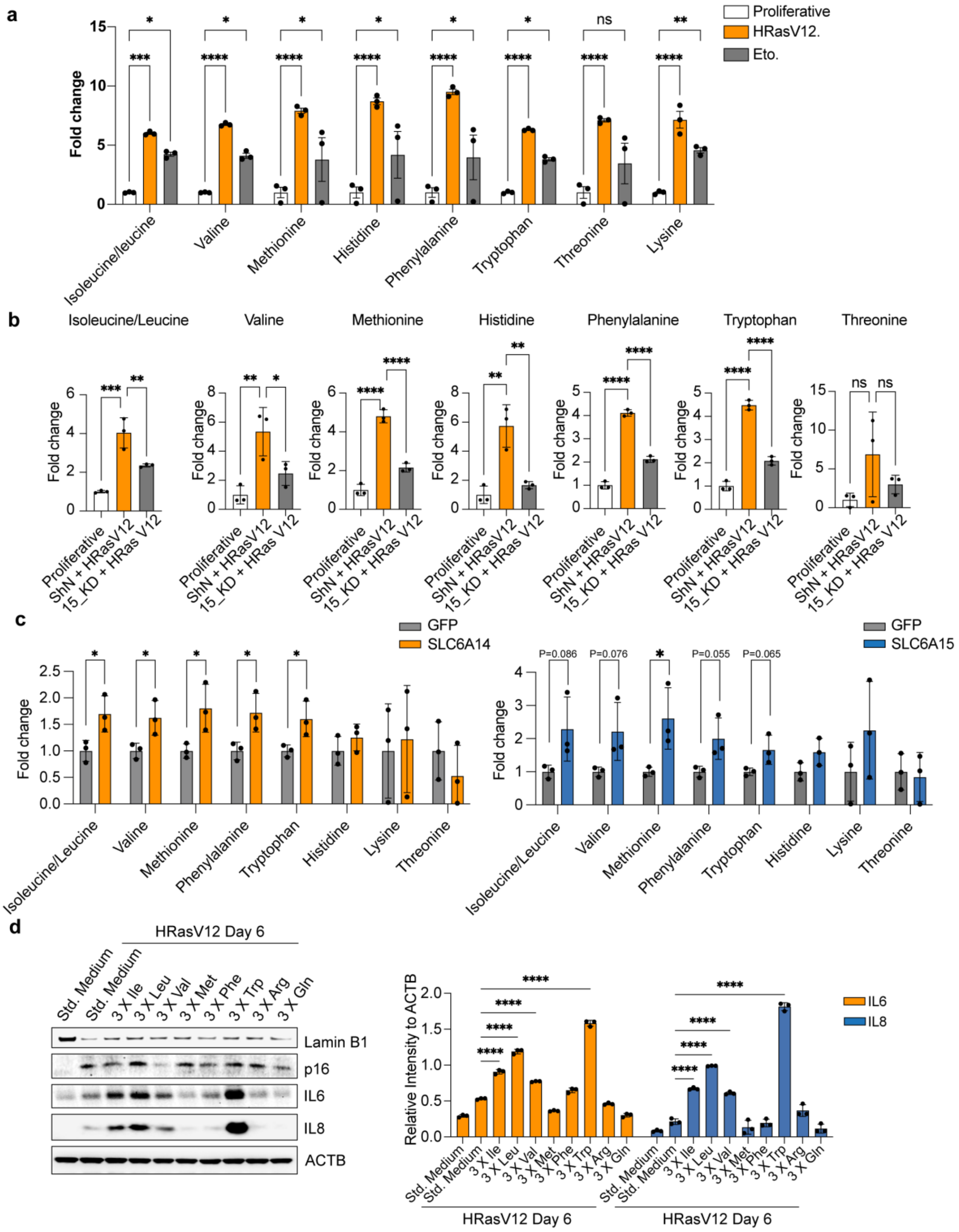

Supplemental Figure 7. Changes in essential amino acid levels in senescent cells. (a) HPLC-MS analysis showing levels of essential amino acids in senescent IMR90 cells. For oncogene-

induced senescence, IMR90 cells with Tet-on HRasV12 were treated with doxycycline (1 µg/mL) for 7 days. For DNA damage-induced senescence, IMR90 cells were treated with 100 µM etoposide for 24 hours and then cultured for 6 days. n=3, mean ± s.e.m., one-way ANOVA test with Dunnett's multiple comparisons. *P < 0.05, **P < 0.01, ***P < 0.001, ****P < 0.0001, ns, not significant. (b) HPLC-MS analysis showing that SLC6A15 knockdown reduces the intracellular levels of multiple essential amino acids in senescent IMR90 cells. (c) HPLC-MS analysis showing changes in essential amino acids in HEK293T cells transfected with SLC6A14 or SLC6A15 for 24 hours. (b-c) n=3, mean ± s.e.m., one-way ANOVA test with Dunnett's multiple comparisons. *P < 0.05, **P < 0.01, ***P < 0.001, ****P < 0.0001, ns, not significant. (d) Immunoblots with its quantitation showed the effects of increasing individual amino acids on SASP factor expression. n=3, mean ± SD, two-way ANOVA test with Tukey's multiple comparisons, ****P < 0.0001. Ile: Isoleucine; Leu: Leucine; Val: Valine; Met: Methionine; Phe: Phenylalanine; Trp: Tryptophan; Arg: Arginine; Gln: Glutamine.

Supplemental Figure 8. SLC6A15 and BCAT1 regulate the expression of multiple SASP factors through mTORC1 signaling pathway.

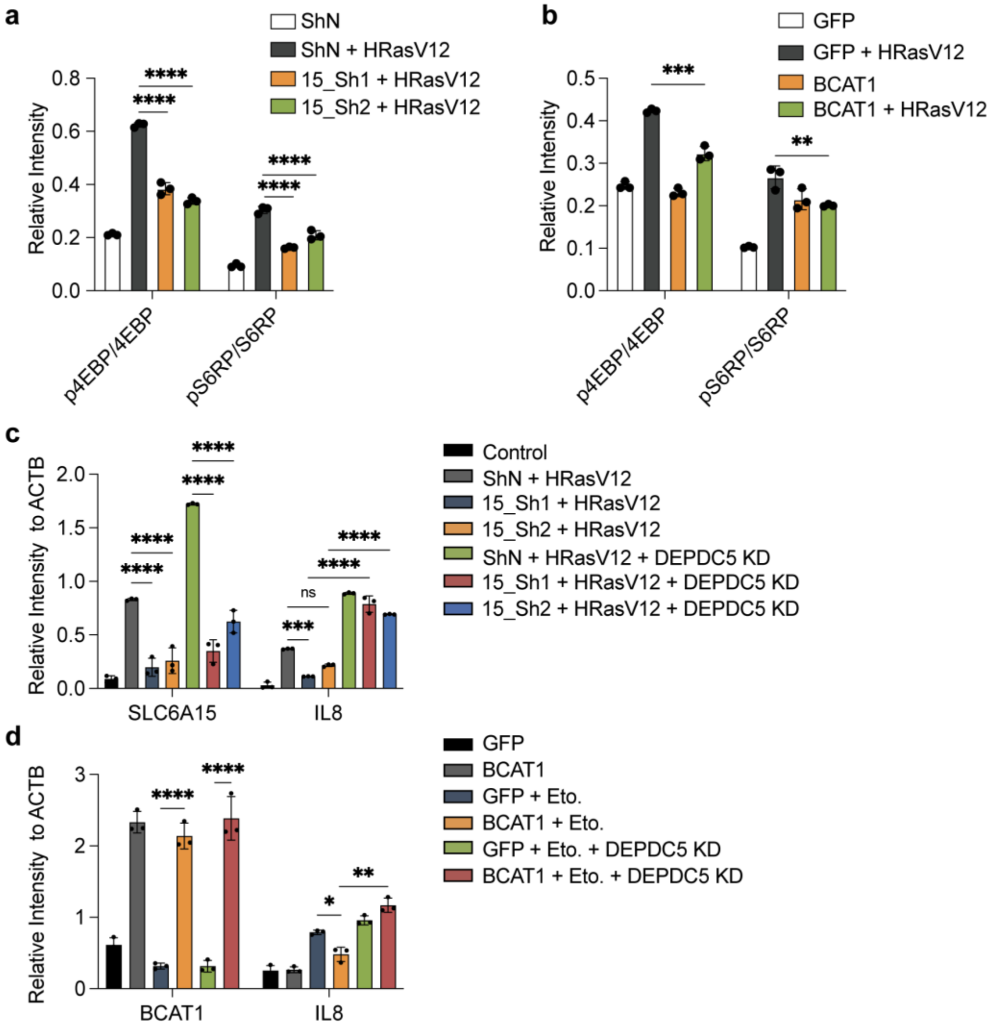

Supplemental Figure 8. SLC6A15 and BCAT1 regulate the expression of multiple SASP factors through mTORC1 signaling pathway. (a-d) Quantitation of Figure 3h (a), 3i (b), 3j (c), 3k (d). n=3, mean ± SD, two-way ANOVA test with Tukey's multiple comparisons $^{**}P < 0.01$, $^{***}P < 0.001$, $^{****}P < 0.0001$.

Supplemental Figure 9. Expression of BCAA regulators is associated with a SASP signature in aged human tissues.

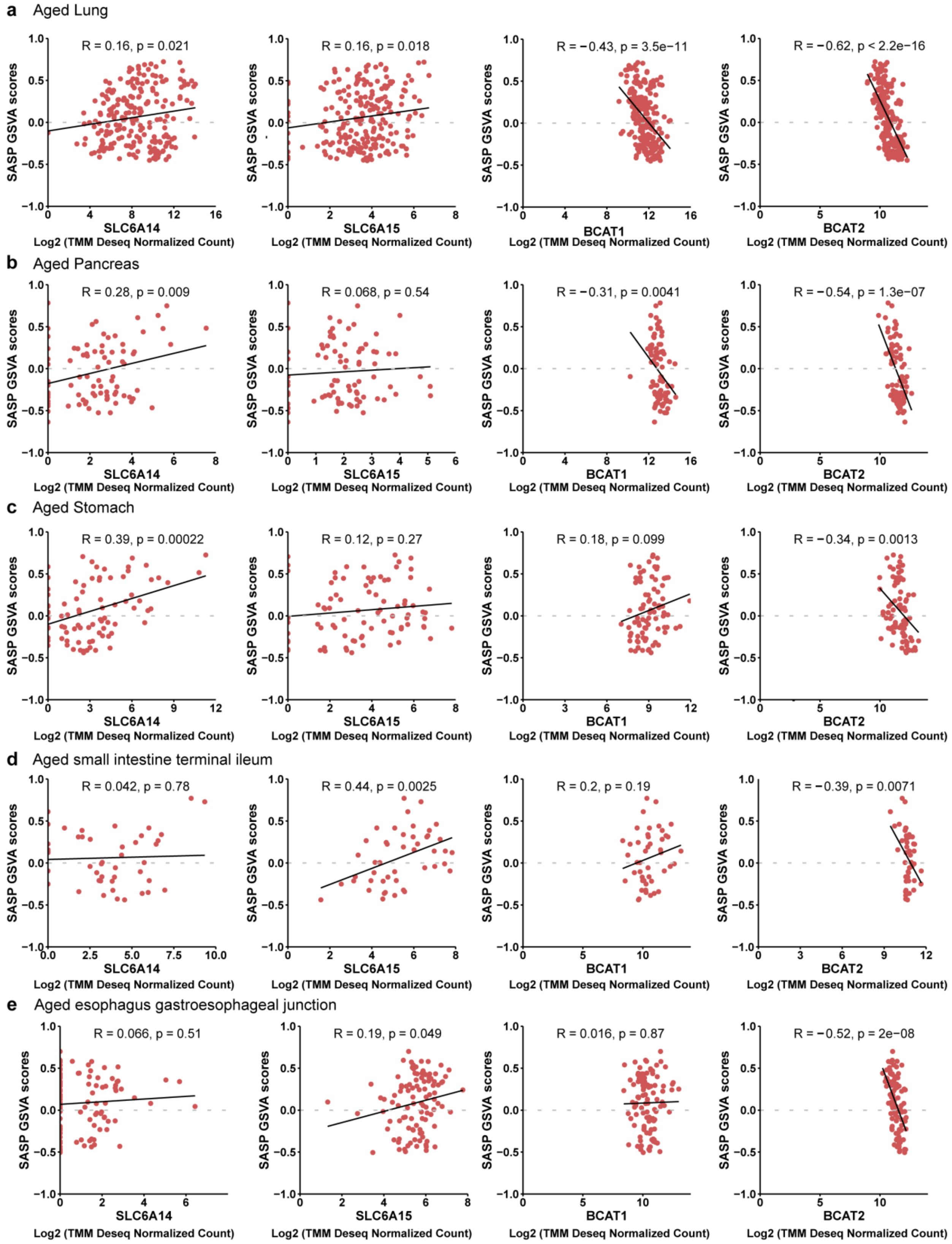

Supplemental Figure 9. Expression of BCAA regulators is associated with a SASP signature in aged human tissues. (a-e) Correlation between SASP signature scores and the expression of BCAA regulators in the indicated tissues from aged humans (60-79 years old).

Supplemental Table 1. Cloning Primers and ShRNA targeted sequence

Cloning Primers	5'-3'
GFP-Mlu-F	GACGCTAGCATCGATACGCGTATGGTGAGCAAGGGCGAGGA
GFP-Spe1-R	ATGCGGATCCTTCGAACTAGTTTACTTGTACAGCTCGTCCATGCC
SLC6A15-Mlu1-F	GACGCTAGCATCGATACGCGTATGCCCAAAAATAGCAAGGTGGT
SLC6A15-Spel-R	ATGCGGATCCTTCGAACTAGTTTACAAATCAGATTCTGGCATATCT
SLC6A14-F-Mlu1	GCTAGCATCGATACGCGTGACAAGTTGAAATGCCCGAG
SLC6A14-R-Spe1	TGCGGATCCTTCGAACTAGTTCATTCCGGTTTTCTGCTGCC
SB-mBCAT1-F	CATCTAGATCTCGAGGAATTCATGAAGGACTGCAGTAATGGATGCTC
SB-GFP-F	CATCTAGATCTCGAGGAATTCATGGTGAGCAAGGGCGAGGAGC
SB-mBCAT1-R	TGTACACTCCGGAAGACGCGTTCAGGGTAGCTCGATTGTCCA
SB-GFP-R	TGTACACTCCGGAAGACGCGTTTACTTGTACAGCTCGTCCATGCC
SB-mSlc6a15sh1-R	TACCCTGAGAGGGCCTATTTCCCATGATTC
SB-mSlc6a15sh1-F	AATAGGCCCTCTTGTGGATGAATACTGCCATTTG
SB-mSlc6a15sh2-F	CATCCACAAGAGGGCCTATTTCCCATGATTC
SB-mSlc6a15sh2-R	TGTACACTCCGGAAGACGCGTTTGTGGATGAATACTGCCATTTG
ShRNA targeted sequence	5'-3'

human_SLC5A15-Sh1	CGTCATCATTGGCTGGAGTTT
human_SLC5A15-Sh2	CCATACCTATGTCAGAAGAAT
Human_BCAT1-Sh1	CCCAATGTGAAGCAGTAGATA
Human_BCAT1-Sh2	CTGCCGGTATTTGACAAAGAA
Human_DEPDC5-Sh1	CCATTGTTCAAGCTCCATAAT
Mouse_Slc6a15-Sh1	CGGCAGCATGTTTGGAACAAT
Mouse_Slc6a15-Sh2	GTGACAATGTTTGACGATTAT

Supplemental Table 2. qPCR primers

Human	Forward primer (5'-3')	Reverse primer (5'-3')
SLC6A14	TGTTTGGTATTTAGCACTTTGTCT	AGGATGAGTAGGACCACATAGG
SLC6A15	AACTGGGCGGGATTGGATTTG	GGTGGCAGAACTTTGTTCACATT
BCAT1	GTGGAGTGGTCCTCAGAGTTT	AGCCAGGGTGCAATGACAG
IL6	AGTGAGGAACAAGCCAGAGC	GTCAGGGGTGGTTATTGCAT
IL8	TCCTGATTTCTGCAGCTCTGT	AAATTTGGGGTGGAAAGGTT
IL1A	ACTGCCCAAGATGAAGACCA	CCGTGAGTTTCCCAGAAGAA
IL1B	AGCTGATGGCCCTAAACAGA	CCTGAAGCCCTTGCTGTAGT
CXLCL1	GAAAGCTTGCCTCAATCCTG	CTTCCTCCTCCCTTCTGGTC
CXCL2	CCCATGGTTAAGAAAATC	CTTCAGGAACAGCCACCAAT
CDKN2A	GTGGACCTGGCTGAGGAG	CTTTCAATCGGGGATGTCTG
CDKN1A	TCACTGTCTTGTACCCTTGTGC	GGCGTTTGGAGTGGTAGA
MMP3	AGGGATTAATGGAGATGCCC	TGAAAGAGACCCAGGGAGTG
MMP1	CCCAAAAGCGTGTGACAGTA	GAGCTCAACTTCCGGGTAGA
A1AT	CCGACTGTTTCCCACACAAG	AGAGAGGTTTGGCGACTTCA
AREG	GAGCCGACTATGACTACTCAGA	TCACTTTCCGTCTTGTTTTGGG
BMP2	ACCCGCTGTCTTCTAGCGT	TTTCAGGCCGAACATGCTGAG
BMP6	AGCGACACCACAAAGAGTTCA	GCTGATGCTCCTGTAAGACTTGA
BTC	CCTGGGTCTAGTGATCCTTCA	CTTTCCGCTTTGATTGTGTGG
CCL2	GCAAGTGTCCCAAAGAAGCT	TCTTGGGTTGTGGAGTGAGT
CCL20	CTCCTGGCTGCTTTGATGTC	ATTTGCGCACACAGACAACT
CCL26	AACTCCGAAACAATTGTGACTCAGCTG	GTAACTCTGGGAGGAAACACCCTCTCC
CCL27	GCAGCATTCCTACTGCCAC	AGGTGAAGCACGAAAGCCTG
CCL3	TGTACCATGACACTCTGCAAC	CAACGATGAATTGGCGTGGAA
CCL8	TGGAGAGCTACACAAGAATCACC	TGGTCCAGATGCTTCATGGAA
CECR1	ACCATGACGAAGAGTGGTCAG	CCATTCGGATGGATTCTGCG
CSF2	TCCTGAACCTGAGTAGAGACAC	TGCTGCTTGTAGTGGCTGG
CSF3	GCTGCTTGAGCCAACTCCATA	GAACGCGGTACGACACCTC
CTGF	CAGCATGGACGTTCGTCTG	AACCACGGTTTGGTCCTTGG
CXCL1	GAAAGCTTGCCTCAATCCTG	CTTCCTCCTCCCTTCTGGTC
CXCL10	CCAAGTGCTGCCGTCATTTTC	GGCTCGCAGGGATGATTTCAA

CXCL2	CCCATGGTTAAGAAAATC	CTTCAGGAACAGCCACCAAT
CXCL3	GCAGGGAATTCACCTCAAGA	TCTCTCCTGTCAGTTGGTGCT
CXCL5	AGCTGCGTTGCGTTTGTTTAC	TGGCGAACACTTGCAGATTAC
CXCL6	GCTCCAAGGTGGAAGTGGTA	AGAAAACTGCTCCGCTGAAG
EREG	GTGATTCCATCATGTATCCCAGG	GCCATTCATGTCAGAGCTACACT
FGF2	AGTGTGTGCTAACCGTTACCT	ACTGCCCAGTTCGTTTCAGTG
FGF7	TCCTGCCAACTTTGCTCTACA	CAGGGCTGGAACAGTTCACAT
GDNF	GGCAGTGCTTCCTAGAAGAGA	AAGACACAACCCCGGTTTTTG
HGF	GCTATCGGGGTAAAGACCTACA	CGTAGCGTACCTCTGGATTGC
ICAM1	AGCGGCTGACGTGTGCAGTAAT	TCTGAGACCTCTGGCTTCGTCA
IGFBP1	TTGGGACGCCATCAGTACCTA	TTGGCTAAACTCTCTACGACTCT
IL10	TCAAGGCGCATGTGAACTCC	GATGTCAAACTCACTCATGGCT
IL11	GCTGCAAGGTCAAGATGGTT	TCTCTCCTTAGCCTCCCTGA
IL11R	CTGGGCTAGGGCATGAACTG	CTGGGACTCCAAGTGCAAGA
IL13	CCTCATGGCGCTTTTGTTGAC	TCTGGTTCTGGGTGATGTTGA
IL19	GGCTCCTGGGTACAATACTGA	GGTGTCCTTAGCTTGGATGGC
IL24	CAGGGTGTGGACAAGGTAACA	CTCAGGATAACATCACGAGTGC
IL33	GTGACGGTGTTGATGGTAAGAT	AGCTCCACAGAGTGTTCCTTG
IL6	AGTGAGGAACAAGCCAGAGC	GTCAGGGGTGGTTATTGCAT
IL7	TTCCTCCCCTGATCCTTGTTC	CTTGCGAGCAGCACGGAATA
IL8	TCCTGATTTCTGCAGCTCTGT	AAATTTGGGGTGGAAAGGTT
KAD3	ACGTGGAAGTCTTCGATGGAG	CACCATGTGTTTCGTAGTCAGA
KITLG	AATCCTCTCGTCAAAACTGAAGG	CCATCTCGCTTATCCAACAATGA
LIF	CCAACGTGACGGACTTCCC	TACACGACTATGCGGTACAGC
LTBP1	CAGCGTGCCTAAACTTTATCAGC	TCAGGAGGATGTTTCACATGGA
MIF	AGAACCGCTCCTACAGCAAGCT	GGAGTTGTTCCAGCCCACATTG
MMP10	TCAGTCTCTCTACGGACCTCC	CAGTGGGATCTTCGCCAAAAATA
MMP12	CATGAACCGTGAGGATGTTGA	GCATGGGCTAGGATTCCACC
MMP13	TCCTGATGTGGGTGAATACAATG	GCCATCGTGAAGTCTGGTAAAAT
NAMPT	ATCCTGTTCCAGGCTATTCTGT	CCCCATATTTTCTCACACGCAT
NRG1	CAGCAGAAGACATGCCAGAG	GTCCACAAATACCCACTTGAGG
PAI2	TCCTGAACCTGAGTAGAGACAC	TGCTGCTTGTAGTGGCTGG
PLAT	AACCCAGATCGAGACTCAAAGC	GGTAGGCTGACCCATTCCC
PLAU	GCTTGTCCAAGAGTGCATGGT	CAGGGCTGGTTCTCGATGG

POSTN	CTCATAGTCGTATCAGGGGTCG	ACACAGTCGTTTTCTGTCCAC
PPBP	GTAACAGTGCGAGACCACTTC	CTTTGCCTTTCGCCAAGTTTC
SIL1	CTGCCTTCATCTAGGATGGCT	GGGTTGGTCAGGGCAAACTC
TFPI2	GTGGCATTCAAGGAGTACCTC	TGATGGCCTTCGATTCTGGATT
TIMP1	AGAGTGTCTGCGGATACTTCC	CCAACAGTGTAGGTCTTGGTG
TNFRSF10C	ACCAACGCTTCCAACAATGAA	CTAGGGCACCTGCTACACTTC
TNFRSF11B	CACAAATTGCAGTGTCTTTGGTC	TCTGCGTTTACTTTGGTGCCA
VEGFA	GGCCAGCACATAGGAGAGAT	ACGCTCCAGGACTTATACCG
GAPDH	ATCAATGGAAATCCCATCACCA	GACTCCACGACGTACTC AGCG
Mouse	Forward primer (5'-3')	Reverse primer (5'-3')
Il6	CCTCTGGTCTTCTGGAGTACC	GTCCTTAGCCACTCCTTCTGT
Il1a	CGAAGACTACAGTTCTGCCATT	GACGTTTCAGAGGTTCTCAGAG
Il1b	GCAACTGTTCCTGAACTCAACT	ATCTTTTGGGGTCCGTCAACT
Tnf	CCTGTAGCCCACGTCGTAG	GGGAGTAGACAAGGTACAACCC
Ptprc	GTTTTCGCTACATGACTGCACA	AGGTTGTCCAACTGACATCTTTC
Nras	TCGCCTGTCCTCATGTATTG	CGCACTGACAATCCAGCTAA
Gapdh	AACTTTGGCATTGTGGAAGG	ACACATTGGGGGTAGGAACA
Slc6a14	GACAGCTTCATCCGAGAACTTC	ATTGCCCAATCCCACTGCAT
Slc6a15	GAATCTGTCAAAGACCTCCTGTC	ATGTATTGCAGCTTACTGTTCCA
Bcat1	TGCCTCTGTTTTGCACTACG	GGTGGAGTAGGGAACCCATT
Fly	Forward primer (5'-3')	Reverse primer (5'-3')
Slc6a15-a	TTCTTCACCATGCTGCTGTC	GCACCTGTGCAGAAGATGAA
Slc6a15-b	GGAATCAAGGTGGTGGAAGA	TCGGGTAGTCCGTACTTTGG
Upd2	CCAAGCCAAATAGAGGACCA	CTTGGTTTGGCAACTGGAAT
AttC	CAACAGTCAGCCGCTTAGTC	GACGTGCCCTGTAGATTCTG
CecA1	TCGCTCTCATTCTGGCCAT	GGTCAACCTCGGGCAGTT
CecA2	TTGGACAATCGGAAGCTGG	TGAGCGATTCCCAGTCCC
CecB	CTTCCTAAGCCAACCAGCCT	ATCAGTCGCACAGTTCTCAC
Mtk	CAATCAATTCCCGCCACCG	CGTATCGAAAATGGGTCCCT
DptA	ACTCCACCACCGCAGTAC	CCTCCCAAGTGCTGTCCATA
DptB	GTGAATCTGCAGCCTGAACC	ATCCCGTGGCATCGAAGG
Dro	CGCCCCATTCGAGTGAGG	GGATGACTTCTCCGCGGTAT
rp49	CCGCTTCAAGGGACAGTATCTG	ATCTCGCCGCAGTAAACGC

Supplemental Table 3. The list of prominent SASP factors for generation of SASP signature.

Number	Gene	
1	BMP6	bone morphogenetic protein 6
2	CCL2	C-C motif chemokine ligand 2
3	CCL20	C-C motif chemokine ligand 20
4	CCL26	C-C motif chemokine ligand 26
5	CCL8	C-C motif chemokine ligand 8
6	CSF2	colony stimulating factor 2
7	CXCL1	C-X-C motif chemokine ligand 1
8	CXCL10	C-X-C motif chemokine ligand 10
9	CXCL2	C-X-C motif chemokine ligand 2
10	CXCL3	C-X-C motif chemokine ligand 3
11	CXCL5	C-X-C motif chemokine ligand 5
12	CXCL6	C-X-C motif chemokine ligand 6
13	EREG	epiregulin
14	HGF	hepatocyte growth factor
15	IL11	interleukin 11
16	IL13	interleukin 13
17	IL15	interleukin 15
18	IL1A	interleukin 1 alpha
19	IL1B	interleukin 1 beta
20	IL33	interleukin 33
21	IL6	interleukin 6
22	IL7	interleukin 7
23	CXCL8	C-X-C motif chemokine 8
24	MMP1	matrix metallopeptidase 1
25	MMP10	matrix metallopeptidase 10
26	MMP13	matrix metallopeptidase 13
27	MMP3	matrix metallopeptidase 3

28	NAMPT	nicotinamide phosphoribosyltransferase
29	SERPINB2	serpin family B member 2
30	TFPI2	tissue factor pathway inhibitor 2
